# Transcriptomic response of *Mytilus coruscus* mantle to acute sea water acidification and shell damage

**DOI:** 10.3389/fphys.2023.1289655

**Published:** 2023-10-26

**Authors:** Zhi Liao, Fei Liu, Ying Wang, Xiaojun Fan, Yingao Li, Jianyu He, Isabella Buttino, Xiaojun Yan, Xiaolin Zhang, Ge Shi

**Affiliations:** ^1^ Laboratory of Marine Biology Protein Engineering, Marine Science and Technical College, Zhejiang Ocean University, Zhoushan, Zhejiang, China; ^2^ Italian Institute for Environmental Protection and Research (ISPRA), Livorno, Italy

**Keywords:** *Mytilus coruscus*, acute acidification, shell damage, biomineralization, shell matrix protein

## Abstract

*Mytilus coruscus* is an economically important marine calcifier living in the Yangtze River estuary sea area, where seasonal fluctuations in natural pH occur owing to freshwater input, resulting in a rapid reduction in seawater pH. In addition, *Mytilus* constantly suffers from shell fracture or injury in the natural environment, and the shell repair mechanisms in mussels have evolved to counteract shell injury. Therefore, we utilized shell-complete and shell-damaged *Mytilus coruscus* in this study and performed transcriptomic analysis of the mantle to investigate whether the expression of mantle-specific genes can be induced by acute seawater acidification and how the mantle responds to acute acidification during the shell repair process. We found that acute acidification induced more differentially expressed genes than shell damage in the mantle, and the biomineralization-related Gene Ontology terms and KEGG pathways were significantly enriched by these DEGs. Most DEGs were upregulated in enriched pathways, indicating the activation of biomineralization-related processes in the mussel mantle under acute acidification. The expression levels of some shell matrix proteins and antimicrobial peptides increased under acute acidification and/or shell damage, suggesting the molecular modulation of the mantle for the preparation and activation of the shell repairing and anti-infection under adverse environmental conditions. In addition, morphological and microstructural analyses were performed for the mantle edge and shell cross-section, and changes in the mantle secretory capacity and shell inner film system induced by the two stressors were observed. Our findings highlight the adaptation of *M. coruscus* in estuarine areas with dramatic fluctuations in pH and may prove instrumental in its ability to survive ocean acidification.

## 1 Introduction

As carbon dioxide (CO_2_) is continuously being released into the atmosphere from various anthropogenic sources and absorbed into surface oceans, it forms carbonic acid in seawater, lowering ambient pH and decreasing the abundance of carbonate ions (CO_3_^2−^) (Sabine et al., 2004; Doney et al., 2009). This process, known as ocean acidification (OA), has far-reaching implications for ocean bio-systems. Thus, OA has emerged as a global environmental issue, and accumulating research has highlighted vulnerabilities in calcification-dependent marine metazoans that are sensitive to changes in carbonate chemistry (Melzner et al., 2020). OA has profound effects on marine organisms and ecosystems, with negative impacts on physiological processes, such as calcification, growth, reproduction, and survival (Strader et al., 2020), in calcifying marine species. Interestingly, some marine species exhibit robust physiological changes relevant to OA (Melzner et al., 2009; Sunday et al., 2014), whereas others are highly sensitive (Teixidó et al., 2018), indicating that the response of bivalves to rising pCO_2_ is species-specific. However, there is still limited mechanistic understanding of the physiological traits responsible for the differential sensitivities.

The genus *Mytilus* represents not only a species with significant economic importance in aquaculture worldwide but also shows strong tolerance to a wide range of environmental factors (Beyer et al., 2017). Previous studies have demonstrated the effects of OA on this species. For instance, the embryonic growth of *Mytilus edulis* appears to be affected by acidified seawater (Bechmann et al., 2011), and the structures of the gills and digestive glands are also affected by seawater acidification, thus inhibiting the energy intake of mussels (Xu et al., 2020). In addition, the calcified shell of *Mytilus* is a crucial protective exoskeleton for this species, and the structural integrity of *Mytilus* shell can be affected by high pCO_2_ (1,000 µatm) even though the biomineralization continues (Fitzer et al., 2014). OA is generally considered to be detrimental to marine shellfishes (Byrne and Fitzer, 2019). The stunting effects of OA on the larval and adult stages of shellfish have been observed in several studies. Reduced seawater CO_3_^2−^ concentration hinders calcification of shellfish, and the acidified seawater can also accelerate the dissolution of CaCO_3_ minerals of calcified shell (Doney et al., 2009; Harv et al., 2018). However, some studies have reported only minor effects of OA on *Mytilus*. For example, in a coupled field and laboratory study, *Mytilus* showed remarkable tolerance to high ambient pCO_2_ when the food supply was abundant (Thomsen et al., 2013). In addition, high rates of *Mytilus* calcifying capacity have been observed even in seawater with highly undersaturated CaCO_3_ (Thomsen and Melzner, 2010), indicating a special biomineralization mechanism of *Mytilus* to combat OA. In *Mytilus*, the mantle is the tissue that produces the shell, and the mature shell is composed of different structural layers, including the nacre and prismatic layers (Gao et al., 2015; Liao et al., 2015). Although *Mytilus* is a globally distributed species with great economic value and ecosystem functions, studies on the effects of OA on *Mytilus* are limited. Several studies have revealed the effects of OA on *Mytilus* physiological functions (Broszeit et al., 2016), larval development (Gazeau et al., 2010; Kelly et al., 2016; Ventura et al., 2016), the byssus (Zhao et al., 2017), and mantle gene expression patterns (Hüning et al., 2013). However, the molecular response, especially the biomineralization-related response of *Mytilus* to OA, remains largely unknown.

As reviewed by Strader et al. (2020), transcriptomics is an effective method and transcriptome profiling is a robust and informative method for examining and elucidating the molecular-level response of marine metazoans to OA. However, the research on the molecular response of *Mytilus* to OA is still limited. Most experiments have been performed with middle- or long-term exposure to OA, ranging from 1 week to over 3 months (Strader et al., 2020). Previous studies have highlighted the long-term adaptation of *Mytilus* to OA (Thomsen et al., 2017), and the ability to continue calcification and maintain *Mytilus* shell integrity under predicted low-pH scenarios has been studied in several key physiological functions of *M. edulis* under OA (Kroeker et al., 2013). Notably, Kapsenberg *et al* recently revealed that some genes involved in the shell development of *Mytilus* larvae exhibited shifts in allele frequencies under low pH conditions, indicating the potential for rapid adaptation to OA in this species (Kapsenberg et al., 2022). To evaluate the molecular responses of *Mytilus* to acute acidification, the mantle transcriptome was sequenced and analyzed for the mussel with a 48 h of exposure time in acidified seawater, and the transcriptomic data of the mantle revealed a rapid gene expressional change. On the other hand, mussels frequently suffer shell fractures or injuries in their natural environment, and therefore shell repair mechanisms in mussels have evolved to counteract shell injury (Hüning et al., 2016). Recently, Yarra et al. (2021) reported a shell repair experimental model for *Mytilus* to explore gene expression patterns during the shell damage repair process, and more than 600 differentially expressed genes were identified under these conditions. However, the shell repair process in mussels with OA remains unclear. Therefore, we performed a transcriptomic analysis of *Mytilus coruscus* mantle with shell damage in normal and acidified seawater. Our data revealed the genes and their functions involved in the rapid response of mussels to OA and provided clues for exploring the biomineralization mechanisms of the mussel mantle under OA and shell damage-repair processes.

## 2 Materials and methods

### 2.1 Mussel sampling and treatment

Adult mussels (70–80 mm in length) were collected from a mussel farm located on the Gouqi Islands of the East China Sea in May 2022. Mussels were acclimated at 22°C for 7 days in clean seawater (pH 8.1 and salinity 19‰). The collected mussels were divided into four groups: mussels with complete shell raised in normal seawater and acidified seawater (designated as CN and CA, respectively), and shell-drilled mussels raised in normal seawater and acidified seawater (designated as DN and DA, respectively). Shell damage model was prepared using the shell drilling method described by Yarra et al. (2021). Shell drilled and undrilled mussels were mixed and raised in normal seawater (pH 8.1) and acidified seawater (pH 7.4), respectively, for 48 h. The pH of the seawater was monitored using a pH meter and controlled by a Seawater acidifier (Starfish SF0S02, Qingdao, China) with a CO_2_ pump. All mussels were fed daily with planktonic food (*Chaetoceros mulleri* and *Isochrysis zhangjiangensis*).

### 2.2 RNA extraction and sequencing

Total RNA was extracted according to the methods used in our previous study (He et al., 2022) from the mantle tissues of the four mussel groups (CN, CA, DN, and DA). In total, there were twelve samples, including three biological replicates for each group, and each sample was collected from six individuals. Complementary DNA (cDNA) from the 12 samples was synthesized using a SuperScript double-stranded cDNA synthesis kit (Invitrogen, Carlsbad, CA, United States of America) with random hexamer primers (Illumina). The cDNA library was then prepared and amplified on cBot (Truseq PE Cluster Kit v3-cBot-HS, Illumina) to generate clusters on the flow cell, which was then paired-end sequenced using a Novaseq 6,000 System (Illumina).

### 2.3 Transcriptomic data processing and bioinformatics analysis

Using the protocol provided in our previous work (He et al., 2022), the raw sequenced reads were trimmed using SeqPrep and Sickle software, quality control was processed using RSeQC (v2.3.6), and the clean reads were separately aligned to the *M. coruscus* genome (release number: PRJEB33342) in the orientation mode using HISAT2 software. The mapped reads were further assembled based on the genome data of *M. coruscus*, and clustered using the Chrysalis clusters software. The “unigenes” were designated by the longest sequences in each cluster. Unigene annotation was performed using BLASTX alignment against NCBI non-redundant protein sequences (NR), Gene Ontology (GO), Eukaryotic Orthologous Groups (KOG), Kyoto Encyclopedia of Genes and Genomes (KEGG), Clusters of Orthologous Groups of proteins (COG), and Pfam databases, with an E-value of <1.0e-5 and the default parameters. The alignment results with the highest homology were used to determine the sequence directions of the unigenes.

To identify differentially expressed genes (DEGs) between the various groups, the expression level of each transcript was calculated according to the transcripts per million reads (TPM) method, and DESeq2 with |log2FC|>1 and Q value ≤0.05 were considered as the threshold for DEGs. In addition, GO and KEGG functional-enrichment analysis were performed at Bonferroni-corrected *p*-value ≤ 0.05 compared with the whole-transcriptome background. GO functional enrichment and KEGG pathway analyses were performed using GoATools and KOBAS online tools, respectively.

### 2.4 Quantitative PCR (qPCR) validation of the RNA-Seq analysis

Using the same RNA samples used in the transcriptomic analysis of the mantle, validation of the RNA-seq data was performed based on the randomly selected 22 DEGs from RNASeq data, following the protocol described in our previous work (He et al., 2022). Briefly, a QuantStudio 1 (Applied Biosystems, Thermo Fisher Scientific) was used for qPCR analysis, and the qPCR cycling conditions were as follows: 95 °C for 10 min, followed by 40 cycles of 95 °C for 15 s and 60°C for 30 s. EF-1α gene was used as an internal control, and the gene expression level was evaluated using the 2^−ΔΔCT^ method. The primers used for qPCR are listed in Supplementary Table S1. A correlation analysis for the expression variation trend of these randomly selected DEGs was performed using Pearson methods.

### 2.5 AB-PAS staining of the mantle edge

The mantle edges from four mussel groups (CN, CA, DN, and DA) were collected and immediately fixed in 4% paraformaldehyde. The tissue samples were dehydrated with a gradient series of ethanol and then embedded in paraffin, and sectioned at 5 μm using a microtome (HistoCore BIOCUT, LEICA). Before histological staining, tissue sections were treated using the protocol described in a previous study (Liu et al., 2019), and the sections were stained with Alcian blue using the periodic acid-Schiff (AB-PAS) method (McMANUS, 1946). Stained sections were examined under a light microscope (ECLIPSE E100; NIKON, Japan).

### 2.6 Transmission electron microscopy (TEM) analysis for the outer fold (OF) in mantle edge and scanning electron microscopy (SEM) analysis for the shell

TEM analysis was performed on the mantle edge according to a previously described protocol (Myers et al., 2007). Briefly, the mantle edges from four mussel groups (CN, CA, DN, and DA) were collected and immediately fixed in a fixative solution containing 4% paraformaldehyde and 2% glutaraldehyde, post-fixed in osmium vapor, and embedded in resin. Sections with thickness of 70–90 nm were cut, floated on copper grids, stained with 2.5% uranyl acetate for 15 min in the dark, and counterstained with lead citrate for 3 min. The sections were viewed and photographed using a Hitachi 600AB transmission microscope (Hitachi, Tokyo, Japan) at an accelerating voltage of 80 kV.

Shells drilled from the mussels under acute acidification were collected and fragmented. The cross-section located at the drilling site was sputter-coated with gold and examined with a SU8010 SEM (Hitachi, Japan) at an accelerating voltage of 3.0 kV.

## 3 Results

### 3.1 Transcriptome sequencing, assembly, and functional annotation

Mantle samples were processed, and 12 cDNA libraries were prepared for the four groups (CN, CA, DN, and DA). The sequencing data after sequencing on a NovaSeq 6,000 System (Illumina) are listed in Supplementary Table S2. Briefly, 72.84 Gb clean data for the 12 libraries were obtained, with an average of 6.07 GB and Q30 > 91% for each library. Of these, more than 97% passed the quality standards and were subjected to further analyses. The transcriptomic data were submitted to the NCBI SRA database (Accession No. of PRJNA918971, and the BioSample names TCN, TCS, TAN, and TAS are in correspondence with CN, DN, CA, and DA, respectively, in this study).

Using HISAT2, the acquired clean reads were mapped to the *M. coruscus* chromosome-level genome (Yang et al., 2021), with an average alignment ratio of 60.75% and an average unique alignment ratio of 57.23% (Supplementary Table S3). Quality Control (QC) of the mapped reads was assessed using RSeQC (v2.3.6), and the coverage of sequencing and the percentage of reads mapped to genome regions are summarized in Supplementary Figure S1 and Supplementary Table S4, respectively. The uniform distribution of reads in the gene body and the high CDS coverage of the reads indicated the high quality of the transcriptomic data. The filtered high-quality reads were assembled into 86, 924 transcripts using Trinity software. The size distribution of all assembled transcripts is shown in Supplementary Figure S2. Most assembled transcripts had a length of >1,800 bp.

All transcripts acquired in this study were annotated against GO, KEGG, COG, NR, SWISS-PROTEIN, and Pfam databases, and a total of 71,437 (82.18%) transcripts were annotated with an E-value threshold of 1 × 10^−5^ (Supplementary Figure S3).

### 3.2 Expression quantity analysis and DEGs

**FIGURE 1:**
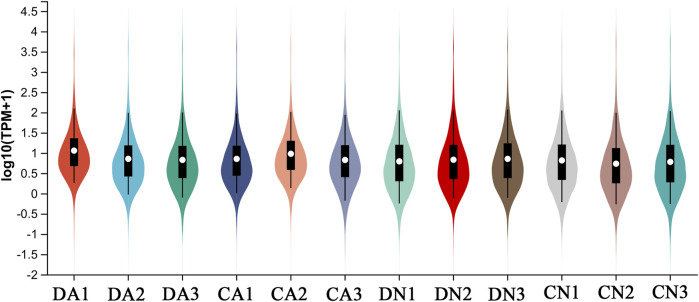
Distribution of expression level (Log10TPM+1) of all transcripts from four groups of mantle samples with triplicate for each group. DA, the mussel with drilled shell and raised in acidified sea water (pH 7.4) with exposure time of 48 h; CA, the mussel with complete shell and raised in acidified sea water (pH 7.4) with exposure time of 48 h; DN, the mussel with drilled shell and raised in normal sea water (pH 8.1); CN, the mussel with complete shell and raised in normal sea water (pH 8.1).

**FIGURE 2:**
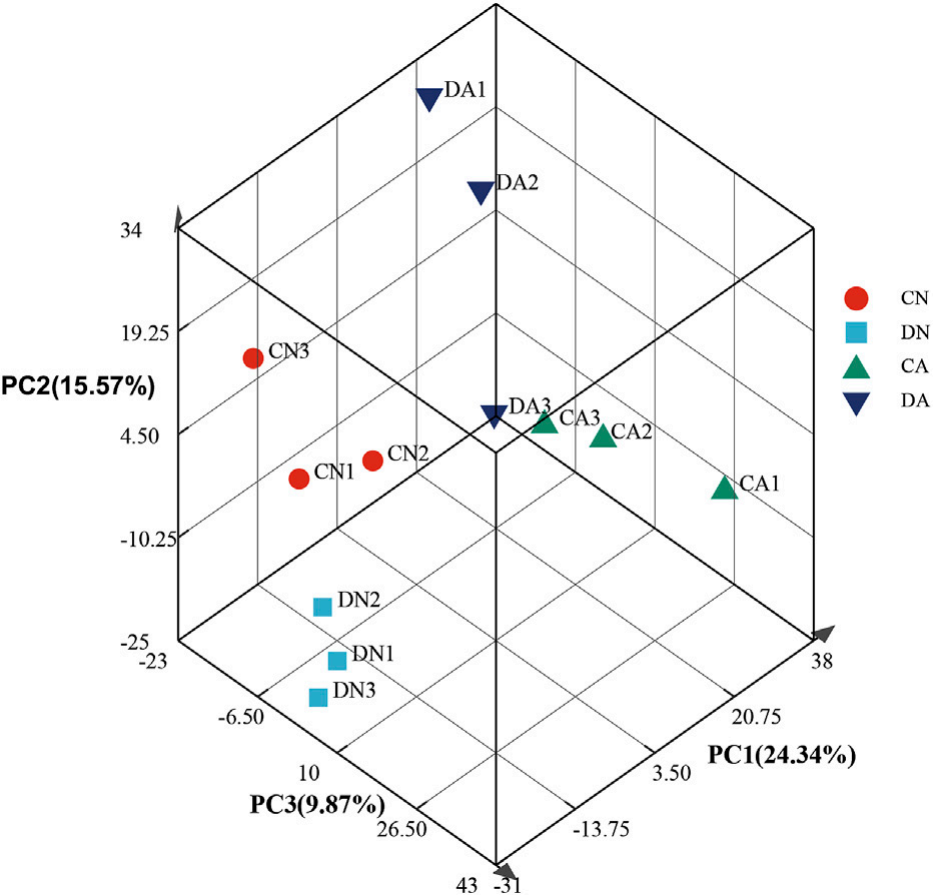
Principle component analysis of gene expression profiles at different phases for the twelve mantle samples. DA, the mussel with drilled shell and raised in acidified sea water (pH 7.4) with exposure time of 48 h; CA, the mussel with complete shell and raised in acidified sea water (pH 7.4) with exposure time of 48 h; DN, the mussel with drilled shell and raised in normal sea water (pH 8.1); CN, the mussel with complete shell and raised in normal sea water (pH 8.1).

The transcriptomes were quantitatively analyzed using RSEM software, and the TPM value was used to assess expression quantity. The expression distribution of all the transcripts is shown in Figure 1. Principal component analysis (PCA) was further performed for the samples, and the three-dimensional PCA score plots (Figure 2) showed that most samples clustered closely and there was an obvious separation between the treated samples (CA, DN, and DA group) and the control samples (CN group), demonstrating the stability and reproducibility of Illumina sequencing. In addition, the correlation between various samples is summarized in Supplementary Figure S4, and the Venn map showing the conserved and unique transcripts with TPM >1 in all samples is summarized in Supplementary Figure S5, showing 17,521 (40.82%) transcripts shared by all the samples.

**FIGURE 3:**
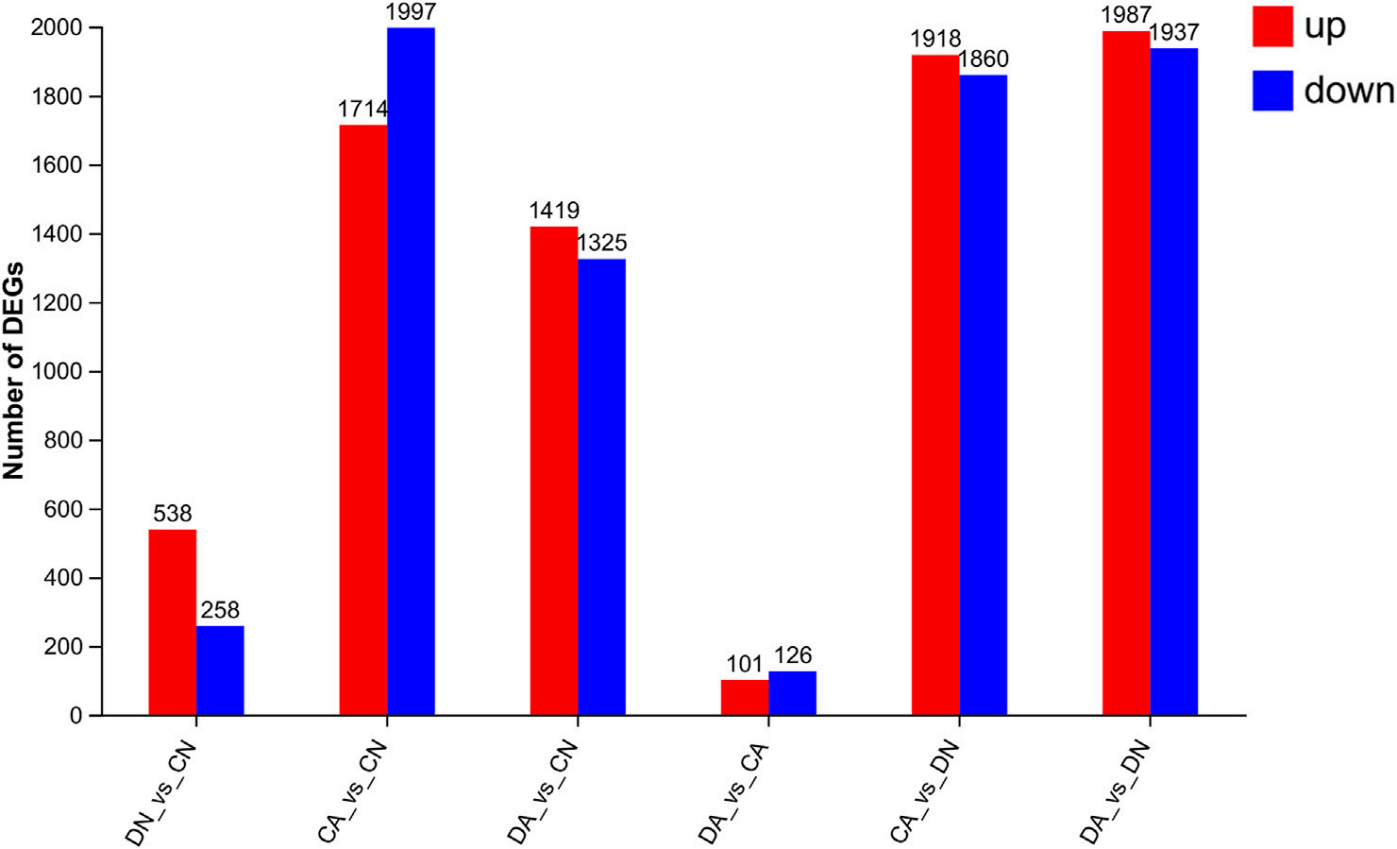
Number of DEGs (fold change > 2, *p*-value < 0.05) from six pairwise comparisons among the mantle samples. DA, the mussel with drilled shell and raised in acidified sea water (pH 7.4) with exposure time of 48 h; CA, the mussel with complete shell and raised in acidified sea water (pH 7.4) with exposure time of 48 h; DN, the mussel with drilled shell and raised in normal sea water (pH 8.1); CN, the mussel with complete shell and raised in normal sea water (pH 8.1).

**FIGURE 4:**
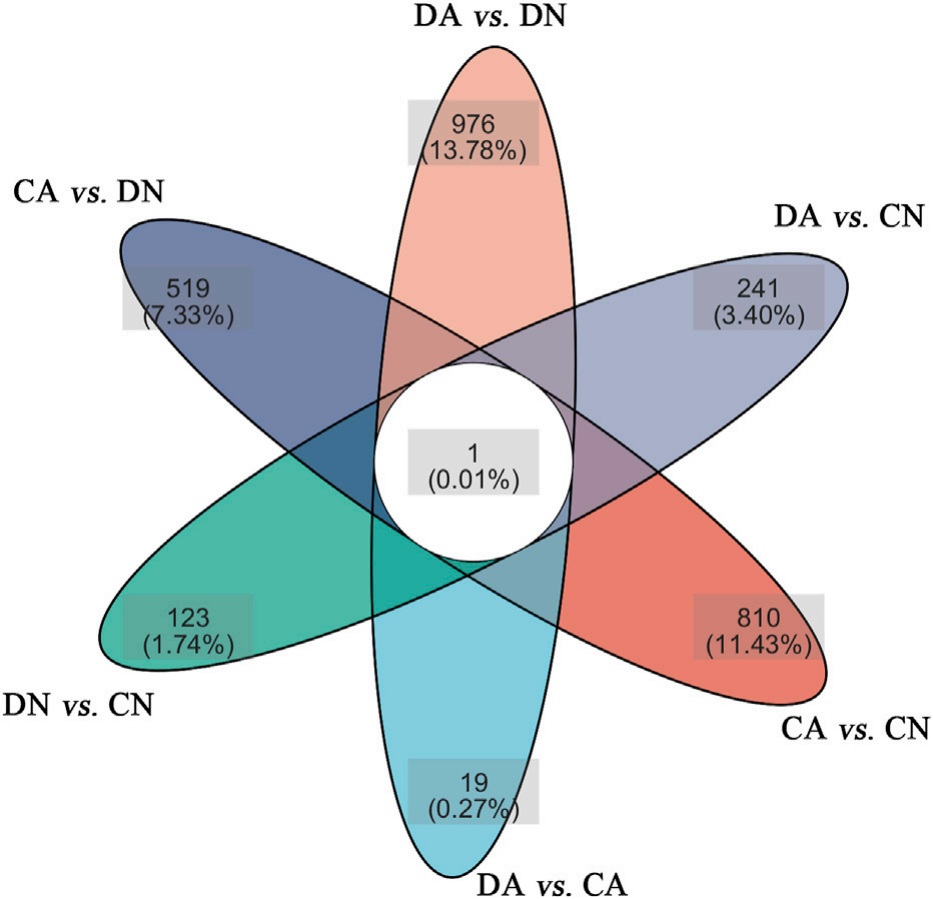
Venn maps of the DEGs in six pairwise comparisons of the mantle samples. DA, the mussel with drilled shell and raised in acidified sea water (pH 7.4) with exposure time of 48 h; CA, the mussel with complete shell and raised in acidified sea water (pH 7.4) with exposure time of 48 h; DN, the mussel with drilled shell and raised in normal sea water (pH 8.1); CN, the mussel with complete shell and raised in normal sea water (pH 8.1).

Using DESeq2 software and Benjamini–Hochberg multiple test calibration method, DEGs with fold change >2 and Q value ≤0.05 were detected from the pair-wise comparisons. Overall, 15,180 DEGs were identified. The majority of DEGs (3,924) were observed in the pairwise comparison of the DA and DN groups, and the minimal DEGs (227) were observed in the DA vs. CA comparison (Figure 3). Venn and volcano maps of these DEGs for the six pairwise comparisons among the four groups are shown in Figure 4 and Supplementary Figure S6, respectively. These results indicated that acidified seawater and shell damage induced completely different responses in the mussel mantle.

### 3.3 Functional annotation and enrichment of DEGs

The results of GO annotation for DEGs of the six pairwise compared groups are summarized in Supplementary Figure S7 with the top 20 GO terms, and detailed information is listed in Supplementary Table S5. The DEGs from the six pairwise comparisons were assigned to 47 GO terms in three categories (molecular function, cellular components, and biological processes). The most dominant GO terms presented in the three categories were “Cellular process,” “Membrane part,” and “Binding,” In addition, DEGs were annotated against the COG database, and the results are shown in Supplementary Figure S8. Most DEGs were annotated as “Function unknown”, followed by “Posttranslational modification, protein turnover, chaperones”, “Intracellular trafficking, secretion, and vesicular transport”,” “Transcription”, and “Signal transduction mechanisms.”

**FIGURE 5:**
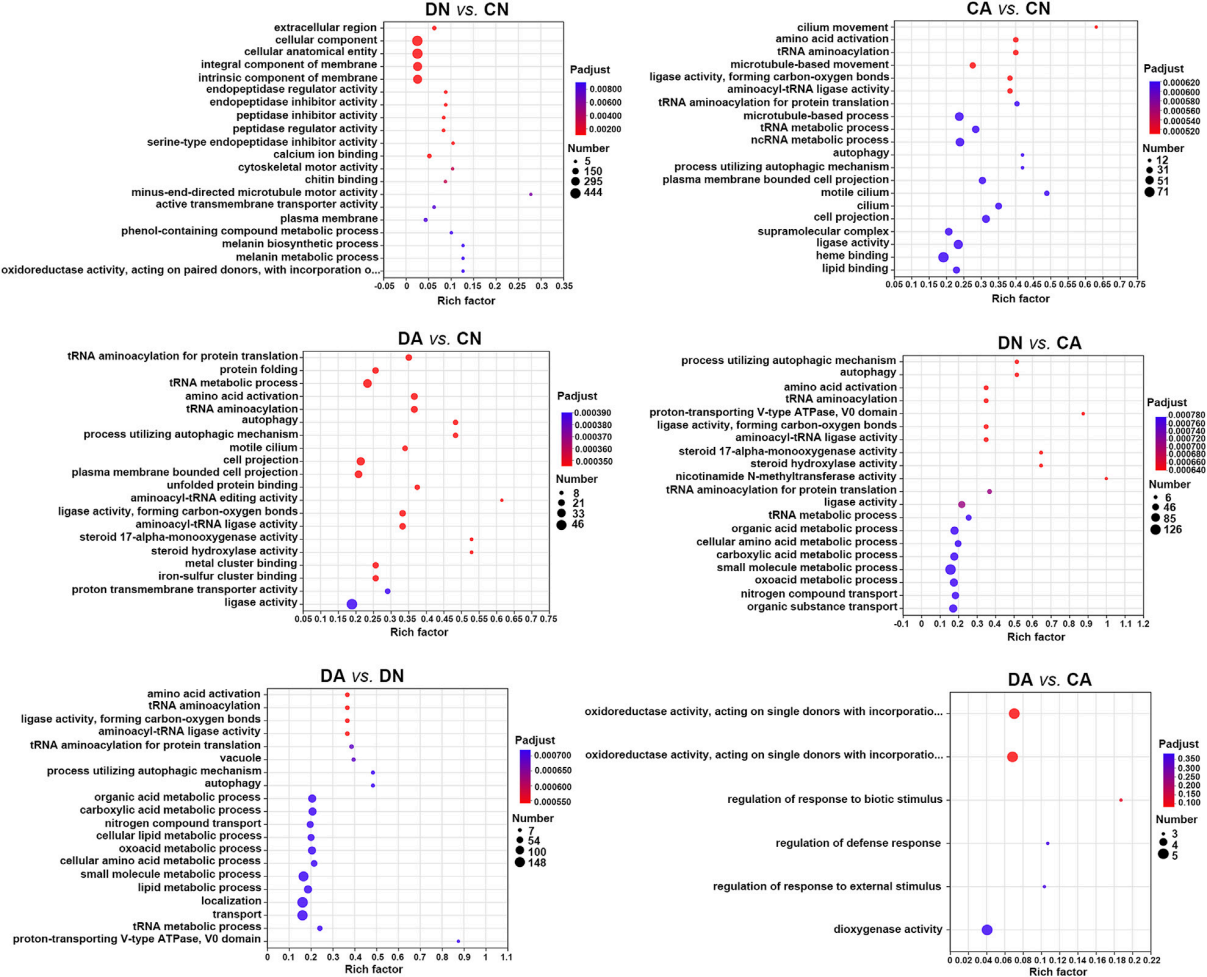
Bubble diagram of top 20 GO terms enriched (with *P*-adjust < 0.05) by DEGs in six pairwise comparisons of mantle samples. DA, the mussel with drilled shell and raised in acidified sea water (pH 7.4) with exposure time of 48 h; CA, the mussel with complete shell and raised in acidified sea water (pH 7.4) with exposure time of 48 h; DN, the mussel with drilled shell and raised in normal sea water (pH 8.1); CN, the mussel with complete shell and raised in normal sea water (pH 8.1). Note that, a *p*-value <0.05 instead of *P*-adjust <0.05 was set as the threshold for the enrichment in DA vs. CA.

**FIGURE 6:**
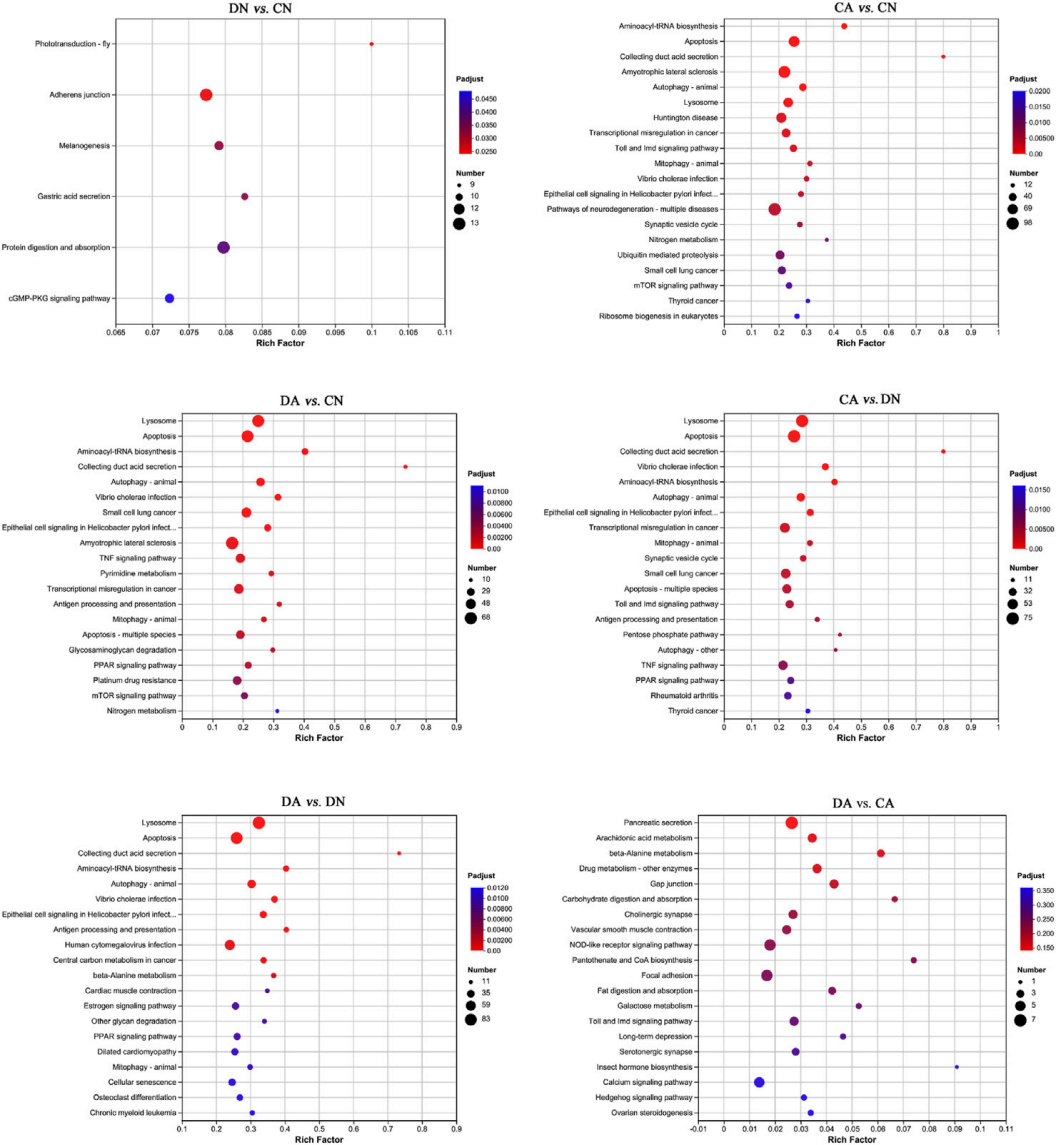
Bubble diagram of top 20 KEGG pathways enriched (with *P*-adjust < 0.05) by DEGs in six pairwise comparisons of mantle samples. DA, the mussel with drilled shell and raised in acidified sea water (pH 7.4) with exposure time of 48 h; CA, the mussel with complete shell and raised in acidified sea water (pH 7.4) with exposure time of 48 h; DN, the mussel with drilled shell and raised in normal sea water (pH 8.1); CN, the mussel with complete shell and raised in normal sea water (pH 8.1). Note that, a *p*-value <0.05 instead of *P*-adjust <0.05 was set as the threshold for the enrichment in DA vs. CA.

Annotated DEGs were subsequently subjected to functional enrichment analysis using Fisher’s exact test (*P*-adjust <0.05), and significantly enriched GO terms and KEGG pathways were identified and summarized as bubble diagrams for the DEGs from six pairwise comparisons (Figures 5, 6). We observed that acute acidification and shell damage induced different GO responses in mussel mantles. As shown in Figure 5, the cellular component, cellular anatomical entity, and integral/intrinsic component of the membrane appeared to be the most enriched DEGs for DN vs. CN, while heme binding, ligase activity, and microtubule-based processes were the most enriched DEGs for CA vs. CN. In addition, the GO terms of small molecule metabolic processes, localization, and transport were the most enriched DEGs in DA vs. DN, indicating the main responses of shell-damaged mussels under acute acidification (Figure 5). Surprisingly, under the same acidified sea water, the comparison of DA vs. CA showed no enriched GO terms with *P*-adjust <0.05 for the DEGs, and a *p*-value < 0.05 was set as threshold and only six GO terms were thus enriched (Figure 5).

The top 20 enriched KEGG pathways for the DEGs of the six paired comparisons are summarized in Figure 6. KEGG enrichment results revealed strong responses in the pathways of adherens junction and protein digestion and absorption in DN vs. CN, pathways of neurodegeneration and amyotrophic lateral sclerosis in CA vs. CN, pathways of lysosomes and apoptosis in DA vs. DN, and pathways of pancreatic secretion and NOD-like receptor signaling in DA vs. CA. No KEGG enrichments with *P*-adjust <0.05 can were acquired for DA vs. CA, and a *p*-value <0.05 was used for the enrichment analysis in this comparison. In addition, the number of upregulated and downregulated DEGs for each enriched KEGG pathway with *P*-adjust <0.05 or *p*-value <0.05 are listed in Table 1. Table 6 shows the 29, 38, 24, 26, and 30 enriched KEGG pathways for DN vs. CN, CA vs. CN, DA vs. CN, DA vs. CA, CA vs. DN, and DA vs. DN, respectively. Most KEGG pathways showed more enriched upregulated DEGs than downregulated DEGs.

**TABLE 1:** KEGG pathways enriched by DEGs in six pairwise comparisons of mantle samples. DA, the mussel with drilled shell and raised in acidified sea water (pH 7.4) with exposure time of 48 h; CA, the mussel with complete shell and raised in acidified sea water (pH 7.4) with exposure time of 48 h; DN, the mussel with drilled shell and raised in normal sea water (pH 8.1); CN, the mussel with complete shell and raised in normal sea water (pH 8.1).

Comparison	Pathway ID	Num	Description	*p*-value	P-adjust	Num of upregulated DEGs	Num of downregulated DEGs
**DN vs. CN**	map04745	9	Phototransduction - fly	2.93E-04	2.57E-02	6	3
	map04520	13	Adherens junction	1.99E-04	2.63E-02	10	3
	map04916	11	Melanogenesis	5.03E-04	3.32E-02	8	3
	map04971	10	Gastric acid secretion	6.40E-04	3.38E-02	6	4
	map04974	13	Protein digestion and absorption	1.47E-04	3.89E-02	9	4
	map04022	11	cGMP-PKG signaling pathway	1.06E-03	4.66E-02	6	5
**CA vs. CN**	map00970	25	Aminoacyl-tRNA biosynthesis	8.92E-09	1.00E-06	25	0
	map04210	75	Apoptosis	3.29E-09	1.11E-06	49	26
	map04966	12	Collecting duct acid secretion	6.95E-09	1.17E-06	12	0
	map05014	91	Amyotrophic lateral sclerosis	1.87E-07	1.58E-05	39	52
	map04140	38	Autophagy - animal	1.16E-06	7.82E-05	35	3
	map04142	60	Lysosome	2.90E-06	1.63E-04	50	10
	map05016	67	Huntington disease	4.24E-05	1.59E-03	28	39
	map05202	51	Transcriptional misregulation in cancer	4.07E-05	1.72E-03	45	6
	map04624	37	Toll and Imd signaling pathway	3.81E-05	1.84E-03	30	7
	map04137	21	Mitophagy - animal	7.18E-05	2.43E-03	21	0
	map05110	22	*Vibrio cholerae* infection	9.42E-05	2.89E-03	14	8
	map05120	25	Epithelial cell signaling in *Helicobacter pylori* infection	1.16E-04	3.27E-03	17	8
	map05022	98	Pathways of neurodegeneration - multiple diseases	1.48E-04	3.84E-03	46	52
	map04721	23	Synaptic vesicle cycle	2.66E-04	6.43E-03	14	9
	map00910	12	Nitrogen metabolism	4.07E-04	9.16E-03	4	8
	map04120	53	Ubiquitin mediated proteolysis	4.59E-04	9.70E-03	41	12
	map05222	45	Small cell lung cancer	5.89E-04	1.17E-02	35	10
	map04150	29	mTOR signaling pathway	7.83E-04	1.47E-02	22	7
	map05216	15	Thyroid cancer	9.89E-04	1.76E-02	14	1
	map03008	20	Ribosome biogenesis in eukaryotes	1.11E-03	1.87E-02	19	1
	map04136	10	Autophagy - other	1.37E-03	2.21E-02	10	0
	map00900	9	Terpenoid backbone biosynthesis	1.52E-03	2.23E-02	8	1
	map04380	23	Osteoclast differentiation	1.50E-03	2.30E-02	18	5
	map04612	14	Antigen processing and presentation	1.93E-03	2.72E-02	12	2
	map04668	42	TNF signaling pathway	2.42E-03	3.15E-02	31	11
	map04910	34	Insulin signaling pathway	2.34E-03	3.17E-02	22	12
	map05418	43	Fluid shear stress and atherosclerosis	3.12E-03	3.90E-02	24	19
	map05230	18	Central carbon metabolism in cancer	3.51E-03	4.23E-02	8	10
	map00330	24	Arginine and proline metabolism	4.19E-03	4.88E-02	12	12
**DA vs. CN**	map04142	64	Lysosome	1.07E-12	3.54E-10	56	8
	map04210	63	Apoptosis	1.40E-09	1.54E-07	34	29
	map00970	23	Aminoacyl-tRNA biosynthesis	1.04E-09	1.73E-07	20	3
	map04966	11	Collecting duct acid secretion	7.21E-09	5.96E-07	10	1
	map04140	34	Autophagy - animal	9.82E-08	6.50E-06	26	8
	map05110	23	*Vibrio cholerae* infection	2.38E-07	1.31E-05	16	7
	map05222	45	Small cell lung cancer	5.19E-07	2.46E-05	20	25
	map05120	25	Epithelial cell signaling in *Helicobacter pylori* infection	8.35E-07	3.46E-05	14	11
	map05014	68	Amyotrophic lateral sclerosis	1.25E-05	4.60E-04	22	46
	map04668	40	TNF signaling pathway	2.63E-05	7.26E-04	19	21
	map00240	17	Pyrimidine metabolism	2.54E-05	7.66E-04	5	12
	map05202	42	Transcriptional misregulation in cancer	3.11E-05	7.92E-04	23	19
	map04612	15	Antigen processing and presentation	2.48E-05	8.19E-04	10	5
	map04137	18	Mitophagy - animal	5.40E-05	1.28E-03	11	7
	map04215	35	Apoptosis - multiple species	8.31E-05	1.83E-03	15	20
	map00531	14	Glycosaminoglycan degradation	1.07E-04	2.09E-03	9	5
	map03320	25	PPAR signaling pathway	1.04E-04	2.14E-03	15	10
	map01524	36	Platinum drug resistance	2.13E-04	3.91E-03	16	20
	map04150	25	mTOR signaling pathway	2.76E-04	4.81E-03	19	6
	map00910	10	Nitrogen metabolism	6.74E-04	1.01E-02	2	8
	map00190	16	Oxidative phosphorylation	7.05E-04	1.01E-02	14	2
	map04152	21	AMPK signaling pathway	6.74E-04	1.06E-02	12	9
	map05216	13	Thyroid cancer	6.54E-04	1.08E-02	12	1
	map04510	54	Focal adhesion	8.14E-04	1.12E-02	15	39
	map04624	27	Toll and Imd signaling pathway	8.82E-04	1.17E-02	13	14
	map00900	8	Terpenoid backbone biosynthesis	1.06E-03	1.30E-02	5	3
	map05323	25	Rheumatoid arthritis	1.06E-03	1.34E-02	22	3
	map04064	41	NF-kappa B signaling pathway	1.38E-03	1.58E-02	21	20
	map04217	45	Necroptosis	1.36E-03	1.60E-02	19	26
	map00983	21	Drug metabolism - other enzymes	2.10E-03	2.32E-02	8	13
	map04120	40	Ubiquitin mediated proteolysis	2.49E-03	2.66E-02	19	21
	map05230	15	Central carbon metabolism in cancer	3.20E-03	3.21E-02	8	7
	map05016	47	Huntington disease	3.11E-03	3.21E-02	15	32
	map04136	8	Autophagy - other	3.36E-03	3.27E-02	7	1
	map05022	71	Pathways of neurodegeneration - multiple diseases	3.72E-03	3.42E-02	27	44
	map04380	18	Osteoclast differentiation	3.62E-03	3.42E-02	14	4
	map00232	4	Caffeine metabolism	4.64E-03	4.15E-02	2	2
	map04211	16	Longevity regulating pathway	5.43E-03	4.73E-02	11	5
**DA vs. CA**	map04972	7	Pancreatic secretion	8.53E-04	1.58E-01	5	2
	map00590	4	Arachidonic acid metabolism	4.61E-03	1.70E-01	3	1
	map00410	3	beta-Alanine metabolism	2.86E-03	1.76E-01	0	3
	map00983	4	Drug metabolism - other enzymes	3.81E-03	1.76E-01	3	1
	map04540	4	Gap junction	2.08E-03	1.92E-01	3	1
	map04973	2	Carbohydrate digestion and absorption	1.30E-02	2.18E-01	1	1
	map04725	4	Cholinergic synapse	1.07E-02	2.21E-01	3	1
	map04270	4	Vascular smooth muscle contraction	1.49E-02	2.29E-01	3	1
	map04621	6	NOD-like receptor signaling pathway	1.28E-02	2.37E-01	1	5
	map00770	2	Pantothenate and CoA biosynthesis	1.06E-02	2.45E-01	0	2
	map04510	6	Focal adhesion	1.75E-02	2.49E-01	1	5
	map04975	3	Fat digestion and absorption	8.09E-03	2.49E-01	3	0
	map00052	2	Galactose metabolism	2.04E-02	2.69E-01	1	1
	map04624	4	Toll and Imd signaling pathway	1.03E-02	2.71E-01	2	2
	map04730	2	Long-term depression	2.57E-02	2.97E-01	1	1
	map04726	3	Serotonergic synapse	2.42E-02	2.99E-01	2	1
	map04913	2	Ovarian steroidogenesis	4.59E-02	3.54E-01	1	1
	map05145	5	Toxoplasmosis	3.70E-02	3.60E-01	1	4
	map00591	2	Linoleic acid metabolism	3.52E-02	3.62E-01	2	0
	map01521	2	EGFR tyrosine kinase inhibitor resistance	4.31E-02	3.63E-01	1	1
	map00232	1	Caffeine metabolism	4.54E-02	3.65E-01	1	0
	map00592	2	alpha-Linolenic acid metabolism	4.18E-02	3.68E-01	2	0
	map05417	6	Lipid and atherosclerosis	3.98E-02	3.68E-01	1	5
	map04927	2	Cortisol synthesis and secretion	3.40E-02	3.70E-01	2	0
**CA vs. DN**	map04142	73	Lysosome	1.59E-11	5.33E-09	65	8
	map04210	75	Apoptosis	2.00E-09	3.36E-07	53	22
	map04966	12	Collecting duct acid secretion	6.10E-09	6.82E-07	0	12
	map05110	27	*Vibrio cholerae* infection	1.38E-07	1.16E-05	19	8
	map00970	23	Aminoacyl-tRNA biosynthesis	1.84E-07	1.23E-05	22	1
	map04140	37	Autophagy - animal	2.45E-06	1.37E-04	36	1
	map05120	28	Epithelial cell signaling in *Helicobacter pylori* infection	3.57E-06	1.71E-04	19	9
	map05202	50	Transcriptional misregulation in cancer	6.19E-05	2.07E-03	42	8
	map04137	21	Mitophagy - animal	6.09E-05	2.27E-03	20	1
	map04721	24	Synaptic vesicle cycle	8.03E-05	2.45E-03	18	6
	map05222	48	Small cell lung cancer	5.96E-05	2.50E-03	37	11
	map04215	42	Apoptosis - multiple species	1.09E-04	3.04E-03	31	11
	map04624	35	Toll and Imd signaling pathway	1.61E-04	3.85E-03	29	6
	map04612	16	Antigen processing and presentation	1.53E-04	3.94E-03	13	3
	map00030	11	Pentose phosphate pathway	1.85E-04	4.12E-03	2	9
	map04136	11	Autophagy - other	2.76E-04	5.77E-03	11	0
	map04668	45	TNF signaling pathway	2.99E-04	5.90E-03	36	9
	map03320	28	PPAR signaling pathway	5.35E-04	9.95E-03	16	12
	map05323	31	Rheumatoid arthritis	6.21E-04	1.10E-02	25	6
	map05216	15	Thyroid cancer	8.80E-04	1.47E-02	14	1
	map00190	18	Oxidative phosphorylation	1.85E-03	2.96E-02	18	0
	map04217	55	Necroptosis	1.95E-03	2.96E-02	37	18
	map00052	12	Galactose metabolism	2.10E-03	3.06E-02	7	5
	map01524	40	Platinum drug resistance	2.46E-03	3.44E-02	30	10
	map00410	14	beta-Alanine metabolism	2.68E-03	3.59E-02	6	8
	map04922	26	Glucagon signaling pathway	2.94E-03	3.78E-02	16	10
**DA vs. DN**	map04142	83	Lysosome	1.16E-14	3.95E-12	76	7
	map04210	76	Apoptosis	1.81E-08	3.08E-06	48	28
	map04966	11	Collecting duct acid secretion	2.62E-07	2.97E-05	11	0
	map00970	23	Aminoacyl-tRNA biosynthesis	6.49E-07	3.68E-05	20	3
	map04140	40	Autophagy - animal	6.43E-07	4.37E-05	33	7
	map05110	27	*Vibrio cholerae* infection	5.71E-07	4.86E-05	19	8
	map05120	30	Epithelial cell signaling in *Helicobacter pylori* infection	1.37E-06	6.67E-05	17	13
	map04612	19	Antigen processing and presentation	5.86E-06	2.49E-04	14	5
	map05163	57	Human cytomegalovirus infection	1.49E-05	5.05E-04	20	37
	map05230	24	Central carbon metabolism in cancer	1.44E-05	5.45E-04	13	11
	map00410	18	beta-Alanine metabolism	4.78E-05	1.48E-03	5	13
	map04260	15	Cardiac muscle contraction	3.88E-04	8.79E-03	5	10
	map04915	31	Estrogen signaling pathway	3.62E-04	8.79E-03	14	17
	map00511	16	Other glycan degradation	3.44E-04	9.00E-03	15	1
	map03320	30	PPAR signaling pathway	3.20E-04	9.07E-03	17	13
	map05414	30	Dilated cardiomyopathy	5.14E-04	1.03E-02	4	26
	map04137	20	Mitophagy - animal	4.86E-04	1.03E-02	16	4
	map04218	32	Cellular senescence	6.27E-04	1.12E-02	16	16
	map04380	25	Osteoclast differentiation	6.04E-04	1.14E-02	17	8
	map05220	18	Chronic myeloid leukemia	6.86E-04	1.17E-02	10	8
	map05200	119	Pathways in cancer	8.45E-04	1.25E-02	61	58
	map04922	29	Glucagon signaling pathway	8.45E-04	1.31E-02	13	16
	map00071	17	Fatty acid degradation	8.12E-04	1.31E-02	7	10
	map05410	29	Hypertrophic cardiomyopathy	9.78E-04	1.33E-02	3	26
	map04152	26	AMPK signaling pathway	9.64E-04	1.37E-02	15	11
	map04212	26	Longevity regulating pathway - worm	1.13E-03	1.47E-02	18	8
	map04022	35	cGMP-PKG signaling pathway	1.30E-03	1.64E-02	13	22
	map00190	19	Oxidative phosphorylation	1.60E-03	1.76E-02	19	0
	map00010	18	Glycolysis/Gluconeogenesis	1.60E-03	1.81E-02	4	14
	map00030	10	Pentose phosphate pathway	1.55E-03	1.81E-02	1	9

### 3.4 Expression levels of representative biomineralization-related and immune-related transcripts

**FIGURE 7:**
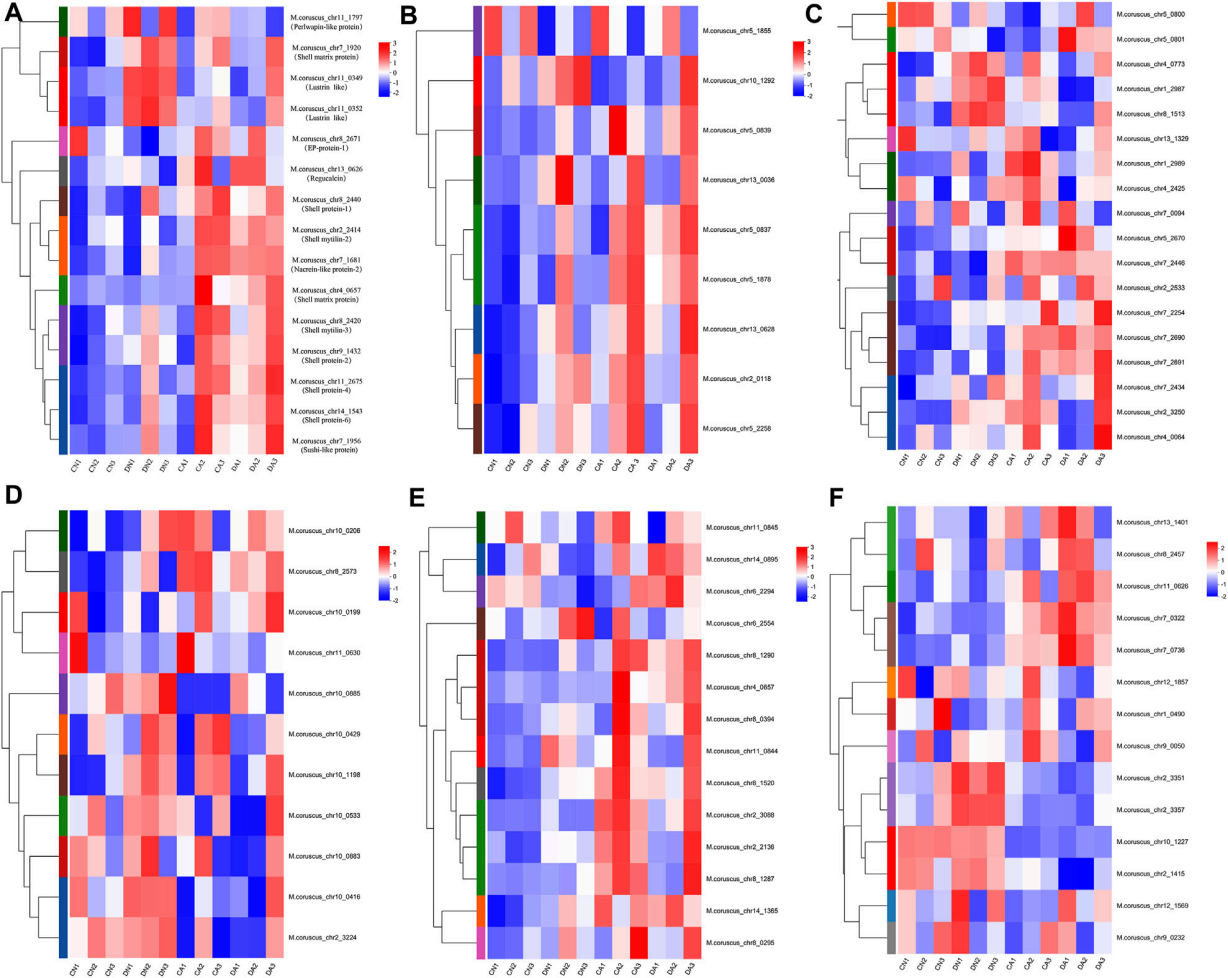
Heatmap of the expression level of transcripts corresponding to the biomineralization-related proteins in each mantle sample. **(A)**, the expressional heatmap for the transcripts of shell proteins, shell matrix proteins, lustrins, nacreins, EP-protein, regucalcin, and perlwapin; **(B)**, the expressional heatmap for the transcripts corresponding to Tyrosinase; **(C)**; the expressional heatmap for the transcripts corresponding to Carbonic anhydrase; **(D)**, the expressional heatmap for the transcripts corresponding to Chitinase; **(E)**, the expressional heatmap for the transcripts corresponding to Chitin synthase; **(F)**, the expressional heatmap for the transcripts corresponding to Perlucin. DA, the mussel with drilled shell and raised in acidified sea water (pH 7.4) with exposure time of 48 h; CA, the mussel with complete shell and raised in acidified sea water (pH 7.4) with exposure time of 48 h; DN, the mussel with drilled shell and raised in normal sea water (pH 8.1); CN, the mussel with complete shell and raised in normal sea water (pH 8.1).

To further explore the biomineralization-related responses of the mussel mantle to different stresses, representative shell matrix proteins (SMPs) were selected based on the shell proteome data of *Mytilus* (Liao et al., 2015). A total of 80 transcripts corresponding to the 12 SMP families were acquired, most of which presented significant changes in TPM value among the four groups. The heatmaps of the expression level for these transcripts are summarized in Figure 7. Detailed information on these transcripts is provided in Supplementary Table S6. The TPM values of the biomineralization-related transcripts showed different expression patterns depending on shell damage, acute acidification, or a combination of the two stresses.

In addition, transcripts of antimicrobial peptide (AMP) and peptidoglycan recognition protein (PGRP) were selected with TPM>0.01 from the mantle transcriptome, and the averaged TPM values of these transcripts in each group are listed in Table 2. Most AMP transcripts showed decreased TPM value under shell damage (DN group) and increased TPM value under acute acidification (CA and DA groups) compared to the TPM value in the CN group. For the PGRP transcripts, three of the five transcripts showed a higher expression level under shell damage (DN group) and acute acidification (CA group) than the TPM value in the CN group. Combined shell damage and acute acidification decreased the expression levels of these PGRP transcripts, with the TPM value being significantly downregulated in the DA group compared to that in the CA group (Table 2).

**TABLE 2:** The averaged expression levels of transcripts of antimicrobial peptides and peptidoglycan recognition proteins in four groups of mantle sample. DA, the mussel with drilled shell and raised in acidified sea water (pH 7.4) with exposure time of 48 h; CA, the mussel with complete shell and raised in acidified sea water (pH 7.4) with exposure time of 48 h; DN, the mussel with drilled shell and raised in normal sea water (pH 8.1); CN, the mussel with complete shell and raised in normal sea water (pH 8.1). The data were statistically analyzed using F test for variance homogeneity and one-way analysis of variance (ANOVA). Significant difference (*p* < 0.05) between the groups was denoted by different letters.

Transcript ID	Acc. No.	Protein description [species]	CN (TPM)	DN (TPM)	CA (TPM)	DA (TPM)
M.coruscus_chr1_1222	ADC29472.1	Mytilin-5 precursor [*Mytilus coruscus*]	1,345.31^a^	511.10^b^	1757.96^a^	8,864.56^c^
M.coruscus_chr1_1224	ADC29471.1	Mytilin-4 precursor [*Mytilus coruscus*]	59.12^a^	49.57^a^	156.55^a^	514.41^b^
M.coruscus_chr1_1225	sp|P81613|	Mytilin-B [*Mytilus edulis*]	65.28^a^	27.08^a^	325.43^a^	1,250.88^b^
M.coruscus_chr9_0955	QHR84800.1	Mytilin 6 [*Mytilus californianus*]	45.25^a^	9.13^b^	69.70^a^	157.58^a^
M.coruscus_chr9_1065	VDI10903.1	Myticin-like [*Mytilus galloprovincialis*]	23.61^a^	20.65^a^	90.72^a^	3,158.54^b^
M.coruscus_chr9_1067	CAC5412204.1	Myticin-like [*Mytilus coruscus*]	7.89^a^	0.71^b^	10.00^a^	46.27^a^
M.coruscus_chr9_1337	sp|P82102|	Myticin-B [*Mytilus galloprovincialis*]	2,795.37^a^	703.59^b^	4,834.70^a^	24,284.83^c^
M.coruscus_chr9_2085	sp|Q86QN6|	Big defensin [*Branchiostoma belcheri*]	32.95^ab^	12.81^a^	58.89^ab^	85.24^b^
M.coruscus_chr5_2960	AFJ04410.1	Myticusin-alpha precursor [*Mytilus coruscus*]	153.58^ab^	53.05^a^	254.78^ab^	791.89^b^
M.coruscus_chr1_3883	AET85055.1	Mytimycin precursor [*Mytilus edulis*]	1.12^a^	0.42^a^	0.57^a^	1.90^a^
M.coruscus_chr13_0961	CAC5407392.1	Peptidoglycan recognition protein [*Mytilus coruscus*]	7.86^a^	23.55^a^	34.84^a^	7.51^a^
M.coruscus_chr13_0962	CAC5407393.1	Peptidoglycan recognition protein [*Mytilus coruscus*]	0.39^a^	2.83^a^	9.01^b^	1.76^a^
M.coruscus_chr14_0979	VDI62795.1	Peptidoglycan recognition protein [*Mytilus galloprovincialis*]	1.23^a^	2.71^a^	16.81^b^	4.23^a^
M.coruscus_chr2_0899	VDI79106.1	Peptidoglycan recognition protein [*Mytilus galloprovincialis*]	4.42^a^	2.49^a^	3.42^a^	5.99^b^
M.coruscus_chr2_0900	CAC5397680.1	Peptidoglycan recognition protein [*Mytilus coruscus*]	0.74^ab^	0.64^a^	0.97^a^	1.79^b^

### 3.5 Validation of DEGs via qRT-PCR

To validate the reliability of the DEG results, 22 transcripts were randomly selected from the DEGs in the mantle, and their expression levels were validated based on their differential expression profiles under the same conditions described in Section 2.1. qPCR was performed for validation, and the relative expressional levels and the correlation between the expression values from qPCR and RNA-seq is shown in Supplementary Figure S9. In most cases, the qPCR results matched the RNA-seq results, and the coefficient of association (*r* value) is 0.656, which means a medium correlation between RNA-Seq and qPCR data. These results indicate that the RNA-seq data reliably identified potential genes under different conditions.

### 3.6 AB-PAS staining of the mantle edge

**FIGURE 8:**
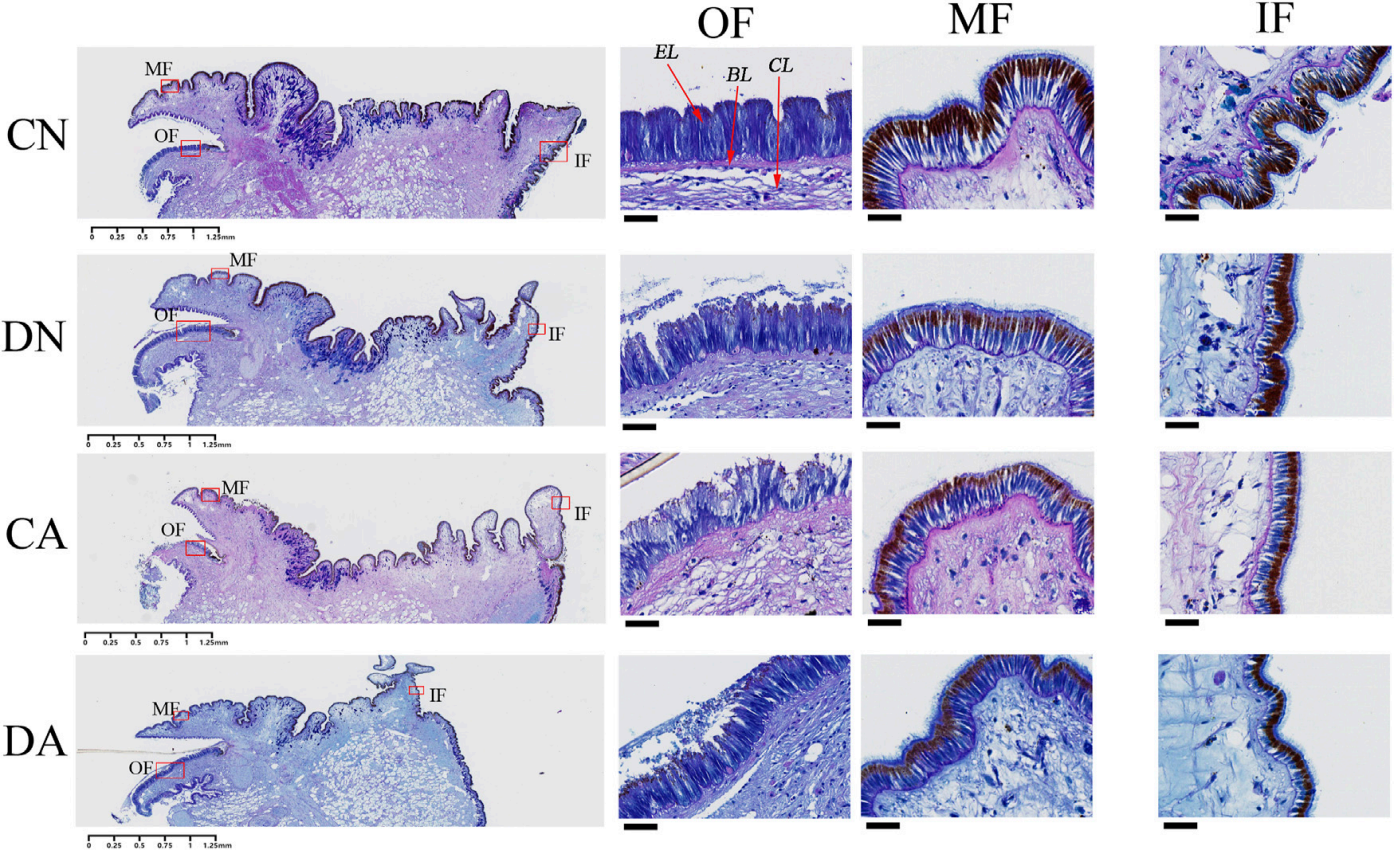
Histological observation of the mantle edge with AB-PAS staining. CN, the mussel with complete shell and raised in normal sea water (pH 8.1); DN, the mussel with drilled shell and raised in normal sea water (pH 8.1); CA, the mussel with complete shell and raised in acidified sea water (pH 7.4) with exposure time of 48 h; DA, the mussel with drilled shell and raised in acidified sea water (pH 7.4) with exposure time of 48 h; OF, outer fold; MF, middle fold; IF, inner fold. EL, epidermis layer; BL, basement layer; CL, connective layer. The left panel represents the whole mantle edge area and the enlarged photos corresponding to the red frames are respectively listed in right three panels. The scar bar is 1.25 mm for the left panel, and 25 μm for the right three panels.

As shown in Figure 8, three folds can be observed in the mantle edge area: inner fold (IF), middle fold (MF), and OF, representing three different regions of the mantle edge. Many invaginations along the mantle epithelium cover the morphogenetic zones of the three folds, and a columnar epithelial layer can be observed on the surface of the three folds (Figure 8). The OF was similar to the MF in terms of length and shape, whereas the IFs were different. As reviewed previously (Kocot et al., 2016; Clark et al., 2020), most bivalve mantles have three folds at the edges. In addition, histological observations revealed an epidermal layer at the surface of the mantle folds, a basement layer under the epidermal layer, and a connective layer under the basement layer (Figure 8). The epidermal layer has been suggested to be a biological regulator of shell formation (Mao et al., 2019; Zhang et al., 2021).

AB-PAS staining also revealed that sulfated and sialic mucosubstances (stained blue) were present mainly in the epidermal layer of the three mantle folds (Figure 8). In addition, neutral mucosubstances (red or purple) were observed mainly in the connective and basement layers of the mantle edge and the epidermis layer of the MFs and IFs. In the OF, both shell damage and acute acidification induced invagination in the epidermal layer and cell morphological changes (Figure 8). Cell damage in the epidermal layer of this fold was observed under both shell damage and acute acidification. Interestingly, the epidermal layers of the MFs and IFs were not affected by either shell damage or acute acidification, as no morphological changes were observed in these two folds (Figure 8). These observations highlight an alteration of the mantle OF epithelium in response to shell damage and acute acidification.

### 3.7 TEM observation of the mantle edge OF

**FIGURE 9:**
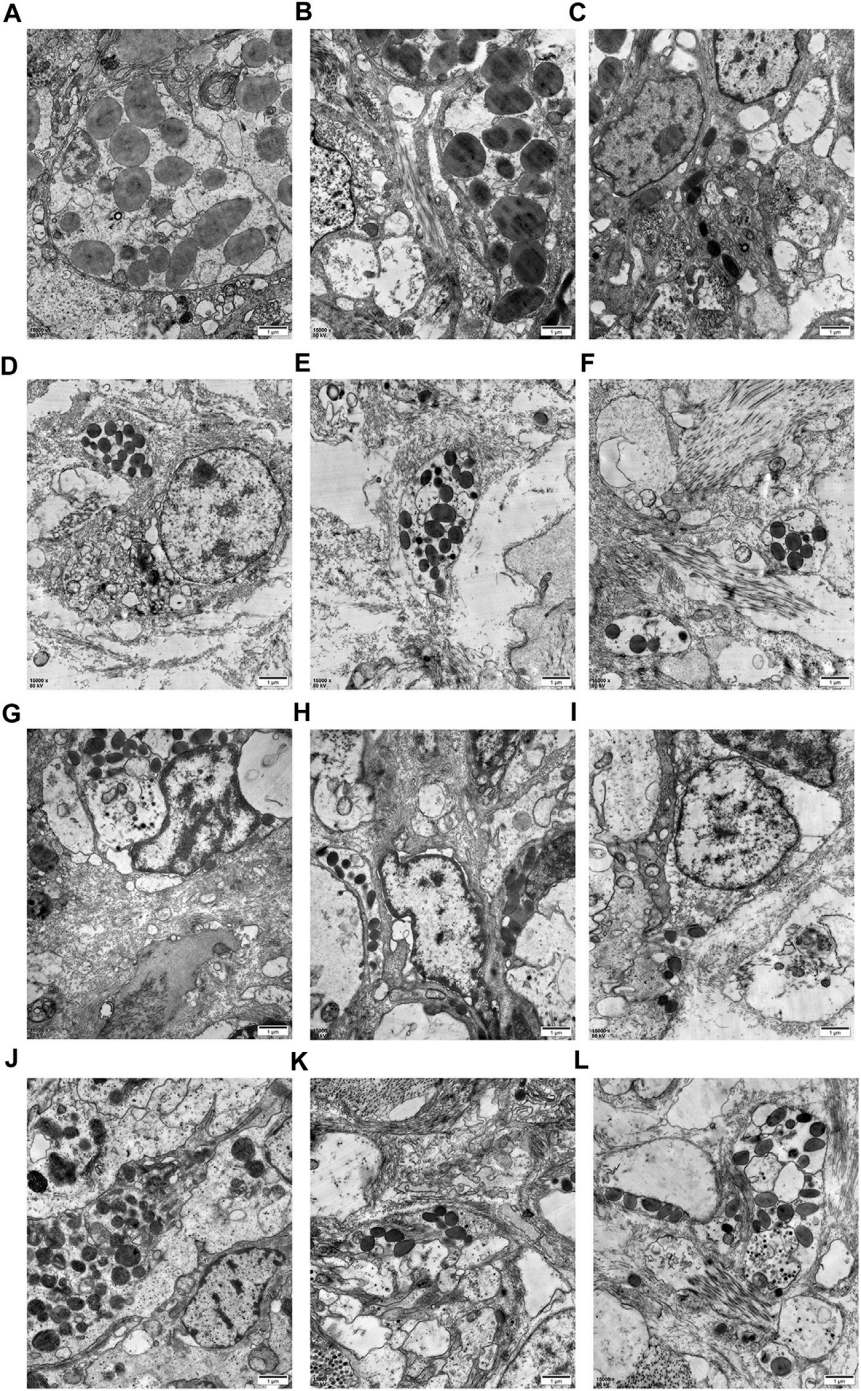
TEM analysis for the secretory cells containing different vesicles or granules in the mantle edge epithelia from the outer fold. (**A–C)**, three duplicate samples from the mussel with complete shell and raised in normal sea water (pH 8.1); (**D–F)**, three duplicate samples from the mussel with drilled shell and raised in normal sea water (pH 8.1); (**G–I)**, three duplicate samples from the mussel with complete shell and raised in acidified sea water (pH 7.4); (**J–L)**, three duplicate samples from the mussel with drilled shell and raised in acidified sea water (pH 7.4).

TEM analysis revealed the presence of different secretory vesicles in mantle epithelial cells. As shown in Figures 9A–C, at least three types of secretory vesicle can be discriminated from the mantle of the control group (CN group), including the vesicle with a diameter of 1–2 μm, electron-lucent core (Figure 9A) with a diameter of 1–2 μm, one or two electron-dense cores surrounded by electron-lucent area (Figure 9B), and vesicles with a diameter of 0.1–0.5 μm and high electron-density (Figure 9C). In the mantle of DN group, vesicles with diameter of 0.2–0.5 μm and high electron-density can be observed as packed tightly in the cells and surrounded by a cytoplasm (Figures 9D–F). In the mantle of the CA group, the number of the observed vesicles was lower than that of other groups, and only some small vesicles with diameter of 0.1–0.4 μm can be observed as packed loosely with or without membrane (Figures 9G–I). While in the mantle of DA group, vesicles with diameter of 0.1–0.8 μm and different electron density can be observed in the mantle epithelia cells (Figures 9J–L). Our results revealed that compared to the CN group, the observed vesicles in the DN, CA, and DA groups were somewhat different in terms of size, electron density, and number, indicating different secretion behaviors of the mantle cells in response to shell damage and/or acute acidification.

### 3.8 SEM observation of the mussel shell under different stresses

**FIGURE 10:**
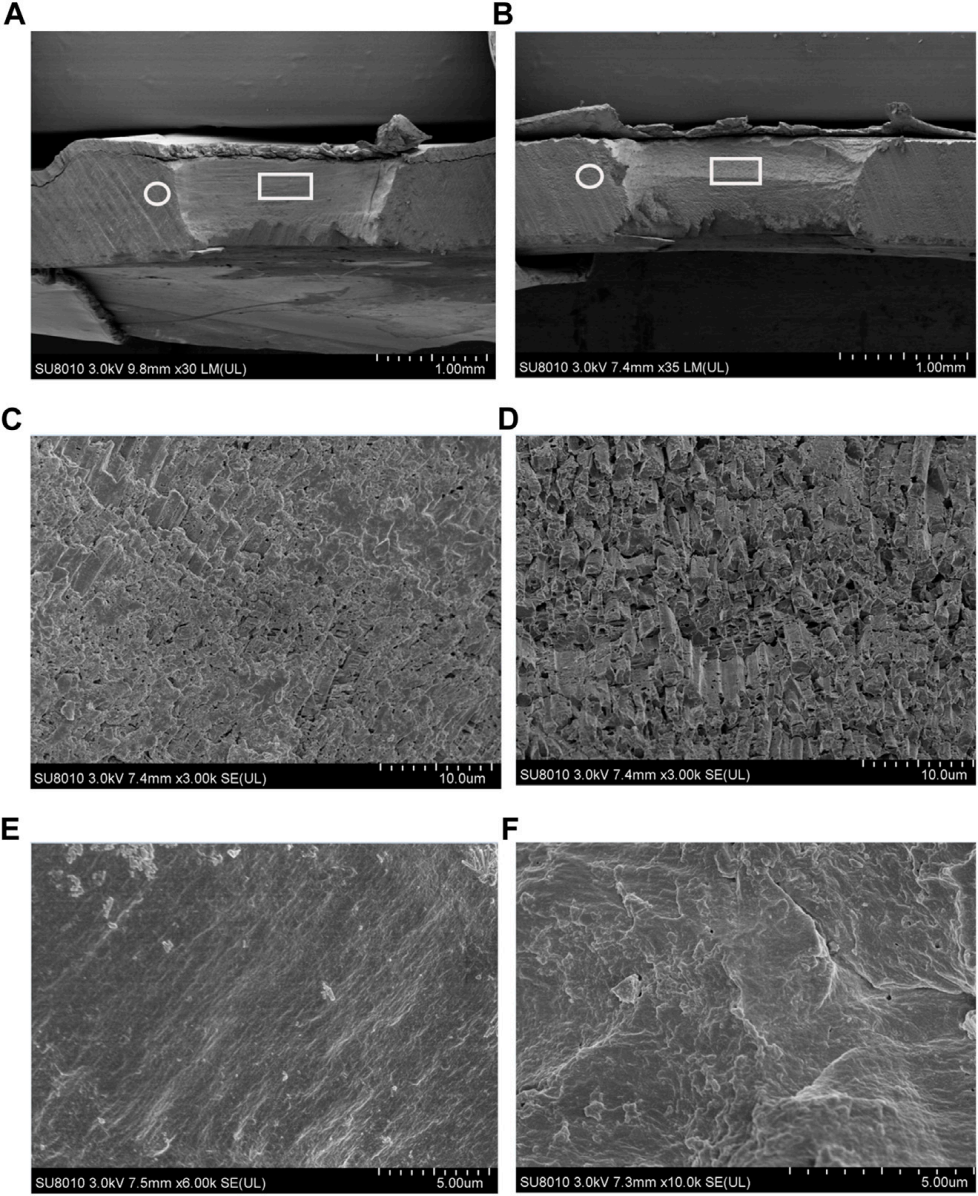
SEM observation of shell cross section located at the drilling site (denoted by a white frame) and the adjacent area (denoted by a white circle). (**A)**, the shell cross section from the mussel under normal sea water (pH 8.1).; (**B)**, the shell cross section from the mussel under acidified sea water (pH 7.4) with exposure time of 48 h; (**C)**, enlargement of the area denoted by a white frame in A; (**D)**, enlargement of the area denoted by a white frame in B; (**E)**, enlargement of the area denoted by a white circle in A; (**F)**, enlargement of the area denoted by a white circle in B.

The cross sections of the shell drilling site and the neighboring area were analyzed via SEM, and the microstructures are presented in Figure 10. Compared to the shell-drilled mussels under normal sea water, acute acidification induced slight textural changes at the shell-drilling site and the neighboring area. At the drilling site, a film was observed on the surface of the cross-section of the shell under normal seawater (Figure 10C), and this film disappeared partially at the drilling site after 48 h of acute acidification (Figure 10D). In the area near the drilling site, a smooth film was observed for the shell under normal seawater (Figure 10E), and thickening of this film with wavy textures was observed for the mussel under acute acidification (Figure 10F). These observations indicate a microstructural change in the inner shell film caused by acidification of the mussel shell structure.

## 4 Discussion

*The M. coruscus* used in this study was collected from the Yangtze River estuary sea area, where seasonal fluctuation of natural pH occurs due to freshwater input; thus, the seawater pH value is reduced rapidly in summer (Wei et al., 2021). This suggests that *M. coruscus* would be impacted if they cannot recover from short-term exposure to coastal acidification. Therefore, short-term exposure of *M. coruscus* to acidification was performed in this study to explore the molecular mechanisms underlying the rapid adaptation of this mussel to coastal acidification.

### 4.1 Functional changes of the mantle cell induced by the stressors

We utilized shell-complete and shell-damaged mussels of *M. coruscus* to investigate whether the expression of mantle-specific genes can be induced by acute seawater acidification and how the mantle responds to acute acidification at the beginning of the shell repair process. According to the number of DEGs between various comparisons, we observed that in the mantle, low pH induced more DEGs than that of shell damage, as 796 and 227 DEGs could be detected in DN vs. CN and DA vs. CA, respectively, whereas more than 3,000 DEGs could be detected in comparisons with changed pH, such as CA vs. CN and DA vs. DN (Figure 3). These results indicate that the mussel mantle is sensitive to acute acidification. GO enrichment analysis further revealed different response patterns of DEGs induced by acute acidification and shell damage. As shown in Figure 5, some reported biomineralization-related GO terms, such as calcium ion binding (Smeets et al., 2015), chitin binding (Furuhashi et al., 2010), and melanin-related processes (Huang et al., 2021), were enriched for DN vs. CN, suggesting rapid activation of the shell repair process, whereas acidification alone induced DEGs to be mainly enriched in metabolic-related processes, such as organic acids, amino acids, and small molecules. Similar results were observed for DA vs. DN and DA vs. CA. Interestingly, no enrichment GO term could be detected with *P*-adjust <0.05 in DA vs. CA, and only five GO terms related to redox reaction and stimuli response regulation were found with a *p*-value <0.35 in this comparison (Figure 5).

KEGG enrichment analysis revealed the responses of mussel mantle to acute acidification and/or shell damage. Under acute acidification, both shell-complete and shell-damaged mussels presented similar responses in the KEGG enrichment analysis, and DEGs from the mussel mantle were mainly enriched in pathways related to cell processes, signal transduction, and metabolism. Apoptosis, lysosomes, and autophagy were apparently the primary enriched KEGG pathways, with the number of upregulated DEGs being much higher than that of downregulated DEGs (Figure 6; Table 1), indicating the activation of these pathways under acute acidification. In *Mytilus*, autophagy, lysosomal function, and mTOR signaling are intrinsically interlinked in response to environmental stress (Sforzini et al., 2018). In marine metazoans, apoptosis is a well-characterized short-term reaction of physiological tolerance to abiotic stress in which damaged cells are removed (Kültz, 2005; Wang et al., 2021). In addition, autophagy is a highly dynamic and conserved pathway that maintains cellular homeostasis via the sequestration and delivery of damaged proteins and organelles into lysosomes for degradation (Parzych and Klionsky, 2014), which is consistent with the activation of the ubiquitin-mediated proteolytic pathway in the comparison of CA vs. CN in this study (Figure 6; Table 1). Lysosomes and mitochondria are decisive controllers of cell death or apoptosis; acidification of lysosomes activates autophagy and triggers apoptosis (Li et al., 2013). Our findings indicate that the pathways of apoptosis, lysosomes, and autophagy in the mussel mantle are induced by acute acidification, and the activation of these pathways may mitigate potential cell damage from reduced pH through the maintenance of cellular homeostasis in the mussel mantle. We also observed that the mTOR signaling pathway, a highly conserved signaling network regulating cell growth in response to nutrients, hormones, and stresses (Wang and Zhang, 2019), was significantly enriched in CA vs. CN, with more upregulated DEGs than downregulated DEGs (Figure 6; Table 1). mTOR has been reported to regulate autophagy, and activation of the mTOR signaling pathway can inhibit autophagy (Al-Bari and Xu, 2020). Surprisingly, in our study, acute acidification induced both the mTOR signaling pathway and autophagy in the mussel mantle. This suggests that in *Mytilus*, the regulation of autophagy may be mTOR-independent and that other signals, such as Ca^2+^ (Al-Bari and Xu, 2020), may play a key role in the activation of autophagy in mantle cells during acute acidification. However, this hypothesis must be verified in future studies.

Shell damage also induced DEGs (DN vs. CN) enriched in the adherens junction, melanogenesis, gastric acid secretion, and cGMP-PKG signaling pathway, with the number of upregulated DEGs being greater than that of downregulated DEGs (Figure 6; Table 1). Adherens junctions play a key role in mediating osteogenesis (Guntur et al., 2012). Melanogenesis is related to the shell pigmentation process, and thus to the formation of the shell periostracum layer (Malachowicz and Wenne, 2019). Gastric acid secretion is an important process for calcium uptake and bone growth in humans (Kopic et al., 2013), and the cGMP-PKG signaling pathway can activate osteoblast function and bone formation in mice (Kalyanaraman et al., 2018). Although the biomineralization-related functions of these KEGG pathways have mainly been reported in the human body or in mice, we cannot exclude the roles of these pathways in shell formation and reparation of *Mytilus*. In addition, other potential biomineralization-related KEGG pathways were detected in the comparisons of CA vs. CN and DA vs. DN, indicating that acute acidification induces a shell formation response in the mussel mantle. For example, collecting duct acid secretion regulates acid–base transport (Wagner et al., 2009) and renal calcification (Moochhala et al., 2008). Amyotrophic lateral sclerosis is another potential biomineralization-related pathway with reported functions in bone metabolism in mice and humans (Joyce et al., 2012; Mizuno et al., 2020). Based on the abovementioned findings, we speculate that different shell repair mechanisms may be adopted by *Mytilus* mantle under normal conditions and in acidified seawater, and the different patterns of enriched biomineralization-related KEGG pathways presented in this study provide clues for exploring specific mechanisms during the shell formation and shell repair processes of mussels under OA.

### 4.2 Morphology and microstructure of the mantle tissue and the shell

Histological observations of the mantle edge highlighted the effects of shell damage and acute acidification on the morphology of the columnar epithelial layer, particularly the OF (Figure 8). Previous studies have revealed that the three folds of the mantle edge are associated with different functions in the bivalve mantle: the IF is involved in water inflow, the MF is involved in sensory functions, and the OF is involved in shell secretion and formation of the periostracum layer (*i.e*., the shell skin) (Clark, 2020; Clark et al., 2020; Salas et al., 2022). In addition, the OF has been suggested to play a critical role in promoting calcium carbonate crystal formation, as many shell matrix proteins have been identified in this fold (Wang et al., 2011; Nakayama et al., 2013). The shell formation of mussel involves the transformation of HCO_3_^−^ to CO_3_^2-^, and a hydrogen ion (H^+^) is thus released (Sillanpää et al., 2018; Sillanpää et al., 2020). Together with the increased H+ generated by OA, the acid–base homeostasis of mantle edge cells may consequently be altered (Ramesh et al., 2020). Therefore, cell morphological changes are understandable for the mantle edge as a cellular response and secretory activity employed by mantle cells to cope with shell damage and/or acute acidification. In this study, we found that epithelial cells covering the mantle edge-stained blue using the AB-PAS technique, indicating that they contain acidic mucosubstances. These acidic mucosubstances may play active roles in the mineralization of mollusk shells by aiding the absorption or binding of calcium ions (Á et al., 2015; Lee et al., 2014). In the mussels under acute acidification, the epithelium of the mantle OF showed fewer AB-PAS-positive cells than the mussels under normal seawater (Figure 8), suggesting a decrease in the secretory activity in the mantle OF and highlighting the negative effect of acute acidification on the shell formation of mussels. TEM observations of the mantle edge presented similar results for the vesicles of mantle secretory cells. The size, electron density, and number of vesicles significantly changed with shell damage and/or acute acidification (Figure 9). However, we found that the vesicles in the shell-damaged mussel mantle were not affected by acute acidification, and even more vesicles could be observed in the mantle cells of the mussel under combined shell damage and acute acidification (Figure 9J-L), suggesting that the mussel tried to start a shell repair process even at a low pH. In addition to the responses of cellular and secretory activity in the mussel mantle under different stressors, further microstructural observation also demonstrated the morphological change of the cross-section at the shell drilling site and the adjacent area, as shown in Figure 10. In this study, we observed that the film covering the prism layer of the shell-drilling site changed, and some erosive holes present on the calcite crystals could be observed in the mussel under acute acidification (Figure 10D). In addition, the smooth film covering the shell underling site became thicker and rougher with a wavy texture presented for the mussel under acute acidification (Figure 10F). These results indicate that acute acidification impairs the shell repair process by affecting the inner shell film and calcite integrity of the prism layer. Disturbed shell ultrastructures in acidified seawater have been previously observed in other *Mytilus* species (Fitzer et al., 2014; Fitzer et al., 2015) and oysters (Fitzer et al., 2018). Although increasing the pCO_2_ had no significant effect on calcite growth, the structural integrity of the shell was disturbed owing to the structural disorientation of the calcite crystals. The inner-shell film system has been reported to be a key component for shell formation and repair in mollusks (Watabe, 1965; Hüning et al., 2016). Morphological changes in the inner-shell film indicate the effects of shell damage and/or acidification on shell biomineralization of *Mytilus*, and the mantle cellular function may thus change in response to stressors.

### 4.3 Expression of shell matrix protein-related transcripts

In *M. coruscus*, a set of 63 SMPs was identified from mussel shells using proteomic techniques (Liao et al., 2015). The genes corresponding to these SMPs were selected from the RNA-Seq data in this study, and the genes with an expression level (TPM value) of >0.01 in each group and are listed in Table 2. A heat map based on this table is summarized in Figure 7. Eighty transcripts corresponding to 12 SMP families were identified, and their expression levels showed complex responses to acute acidification and/or shell damage. Shell proteins, such as shell proteins −1, −2, −4, and −6, were induced with increasing TPM values by acute acidification or shell damage (Figure 7A). Similar results were observed for nacrein, sushi-like protein, EP protein, and regucalcin (Figure 7A), which are all members of the proposed universal molluscan biomineralization-related proteins (Arivalagan et al., 2017). We also noted that nacrein and lustrin, both SMPs involved in shell nacre layer formation (She et al., 1997; Song et al., 2014), presented much higher expression levels under acute acidification than those under shell damage alone, suggesting a compensatory response of these two SMPs to counter OA. In addition, most transcripts of tyrosinase also presented similar changes (Figure 7B), as a high TPM value for this SMP was observed under acute acidification alone or combined with shell damage, which implied vital roles of tyrosinase in the formation and repair of mussel shells under OA. Similar results were observed for *Crassostrea angulata* larvae. The mRNA level of tyrosinase was significantly upregulated in early d-veliger larvae in the 3,000 ppm CO_2_ treatment group (Yang et al., 2017). However, in another case, the expression of the tyrosinase gene was significantly downregulated in Pacific oyster *Crassostrea gigas* larvae in acidified seawater (Liu et al., 2020). This may be due to the different sensitivities of these two closely related *Crassostrea* species to low pH stress, as described in a previous study (Moreira et al., 2016). Carbonic anhydrase (CA) is another SMP involving in biomineralization, playing a key role in CO_2_/HCO_3_^−^ conversion for CaCO_3_ precipitation of *Bivalvia* shell (Roleda et al., 2012). The crystallographic control and CaCO_3_ mineral growth may be disrupted if OA inhibits CA (Fitzer et al., 2014). In this study, 18 transcripts of CA were identified, most of which were upregulated under acute acidification and/or shell damage (Figure 7C), suggesting rapid activation of CA to counter OA and shell damage in the mussel mantle. Similar results were reported in the case of *Crassostrea* species, and increasing CA activity has been proposed as a high tolerance capacity of the oyster to the tested conditions (Moreira et al., 2016). However, we also noted that several CA transcripts, such as *M. coruscus* _chr13_1329 and *M. coruscus* _chr5_0800, were downregulated under low pH and/or shell damage conditions (Figure 7C; Table 2), suggesting that the role of CA in mussel shell biomineralization is complex. Considering the role of chitin in the shell composition of *Mytilus* and snails (Schönitzer and Weiss, 2007; Yonezawa et al., 2016), chitinase and chitin synthase are the two key enzymes that regulate shell chitin metabolism and provide a chitin layer inside the shell during growth. Both chitinases and chitin synthases have been identified in *M. coruscus* (Liao et al., 2015). In this study, most transcripts of both chitinase and chitin synthase were upregulated under shell-damage stress (Figures 7D,E), suggesting critical roles for these two enzymes in the shell-repair process. Similar results were observed in a previous study (Yarra et al., 2021). Interestingly, acute acidification inhibited the expression of most chitinase (Figure 7D; Table 2) and upregulated the expression of most chitin synthase transcripts (Figure 7E; Table 2). This indicates that *Mytilus* may simultaneously promote chitin synthesis and inhibit chitin degradation to maintain the integrity of the chitin shell, thus mitigating the impairments caused by OA. Perlucin is a lectin C-type domain-containing SMP with a reported function in calcium carbonate crystallization in mussel shells (Blank et al., 2003). In this study, the expression level of most perlucin transcripts in the mussel mantle showed a complex response, as some perlucin transcripts showed inhibition by shell damage and activation by combined shell damage and acute acidification, whereas others showed a reverse trend (Figure 7F; Table 2). This indicates different functions of different perlucin transcripts under different stressors. Perlucin has been reported to be involved in the innate immune defense in shrimp and abalones (Bi et al., 2020; Choi et al., 2022). Considering the multiple functions of perlucin in both biomineralization and innate immunity, we speculate that some perlucins may participate in innate immune defense, while others are involved in shell formation in *Mytilus*, and different responses of different perlucin transcripts to acute acidification and shell damage may reflect the complex roles of this molecule.

### 4.4 Expression of immune-related and energy-related transcripts

Notably, the mantle is not only a tissue for shell formation but also an immune-related tissue in the mussel innate immune system (Huang et al., 2022; Li et al., 2022). Previous studies have demonstrated that elevated pCO_2_ has a significant effect on the immune system of Pacific oysters, with the mRNA expression patterns of several immune-related genes varying depending on the exposure time and tissues (Wang et al., 2016). In addition, the upregulation of C-type lectin was observed in *Mytilus* under long-term exposure to high pCO_2_, indicating that pCO_2_-driven OA may trigger specific immune-related genes (Castillo et al., 2017). In the present study, immune-related pathways in the mantle were significantly affected by acute acidification, particularly in mussels with shell damage. We noted that the pathways of Toll and Imd signaling, *V. cholerae* infection, and ubiquitin-mediated proteolysis were significantly enriched in the CA vs. CN comparison (Figure 6). In *Mytilus*, Toll signal transduction pathway is an important immune-related pathway that can be triggered by either Gram-positive or Gram-negative bacteria (Castillo et al., 2017). The *Vibrio cholerae* infection pathway is enriched in the mussel gills and gonads under bacterial stress (He et al., 2022), indicating an immune-related function of this pathway in *Mytilus*. Interestingly, for the shell-damaged mussels, extra immune-related pathways were detected under acute acidification, as enriched TNF signaling and antigen processing and presentation were observed in the DA vs. CN comparison. The function of the TNF signaling pathway in *Mytilus* immunity has been confirmed in previous studies (Betti et al., 2006; Toubiana et al., 2014; Qi et al., 2018), and enrichment of this pathway in bacteria-induced mussels has also been detected in the gills and gonads of *Mytilus* (He et al., 2022). Interestingly, no immune-related KEGG pathway was enriched in the comparison of DN vs. CN, suggesting that shell damage stress alone did not induce a strong immune response in the mussel mantle. In addition, most AMP-related transcripts were downregulated under shell-damaged conditions, indicating immune suppression in shell-damaged mussels. Interestingly, acute acidification induced a rapid upregulation of these AMP transcripts, including mytilin, myticin, big defensin, and myticusin, and the highest TPM value was observed in mussels with combined acute acidification and shell damage (Table 2), suggesting immunomodulation following acute acidification exposure, and a preparation of the mantle for possible subsequent infection under OA. The expression pattern of PGRPs showed a slightly different response to that of AMPs. Of the five PGRP transcripts, three were upregulated and two were downregulated in response to shell damage alone, and acute acidification induced high TPM values in these PGRPs. These results are partially consistent with those reported previously, as pCO2-driven OA promotes the transcription of several AMPs and PGRP in mussel gills (Castillo et al., 2017).

Previous studies have revealed the effects of OA on energy-related metabolism in marine metazoans, and metabolic depression is commonly observed with the downregulation of genes associated with ATP production in response to OA (Hüning et al., 2013). However, some species with high tolerance to OA have adapted to constitutively express genes that enable high ATP production (Evans et al., 2017). These studies highlighted that the core response of marine metazoans to a decrease in pH involves reallocation and/or changes in the production of ATP, often as a trade-off in maintaining ion homeostasis, calcification, and control of internal pH levels (Strader et al., 2020). In the present study, we observed that only a few energy-related pathways in the mussel mantle were affected by low pH stress. For example, oxidative phosphorylation, fat digestion and absorption, and glycolysis/gluconeogenesis were enriched in the comparisons of DA vs. CN, DA vs. CA, and DA vs. DN (Table 1). Notably, the most enriched DEGs in the oxidative phosphorylation pathway were upregulated under acute acidification, indicating increased ATP production capacity in the mussel mantle in response to acute acidification. This finding suggests that mussels invest more energy in acclimatizing to acute acidification via the activation of oxidative phosphorylation. Similar results have been reported for other species with a high tolerance to OA (Davies et al., 2016; Evans et al., 2017). In addition, a previous study revealed that the concentrations of ATP and ADP in the adductor muscle of *M. edulis* showed no significant changes in response to high pCO_2_, indicating the tolerance of *Mytilus* to OA (Matoo et al., 2021). Our results partially confirm this view, and the elevated oxidative phosphorylation and upregulated gene expression of AMPs and biomineralization-related genes may represent positive responses of *M. coruscus* adapted to OA resistance. Taken together, our study provides important data on the extant levels of plasticity in the mantle transcriptome of mussels, as well as insights into the potential adaptation of mussels to future global change.

## 5 Conclusion

In the present study, we conducted a transcriptomic analysis of the bivalve *M. coruscus* under various conditions to ascertain the molecular responses of the mussel mantle to different stressors and comment on the potential adaptation strategy of *M. coruscus* in estuarine conditions that naturally experience low pH and frequent shell breakage. Here, we report the different gene expression patterns of the mussel mantle under different stresses (shell damage, acute acidification, and a combination of these two stressors), showing a strong response in the mussel mantle to acute acidification. Functional analysis of the DEGs from various comparisons in this study revealed that *M. coruscus* appear to improve its ability to continue biomineralization under OA by activating biomineralization-related pathways and upregulating the expression levels of some SMPs and AMPs, which suggests tolerance to acute acidification and a capacity to maintain the shell-repair process even under high pCO_2_. It should be noted that other genes with reported potential function in shell-repairing are not significantly modulated in this study, such as genes with function in proteases and protease inhibitors, methyl translation, cell signalling, and ion balance (Yarra et al., 2021). Considering the differences of our study and other studies in the species and the exposure time of mussel to acidification and shell-damage, our study revealed the earlier gene modulation of mussel mantle more than that of whole shell-repairing process under normal or acidified seawater. Morphological analysis further highlighted the importance of the OF at the mantle edge in the shell repair process. Microstructural analysis revealed changes in *M. coruscus* mantle cells and the shell cross-section under shell damage and/or acute acidification, implying increased secretory activity and a cost to the structural integrity of the shell for maintaining shell formation and repair under acute acidification. Our findings highlight the adaptation of *M. coruscus* in estuarine areas with dramatic fluctuations in pH and may prove instrumental in its ability to survive OA. However, to project the future implications of OA on the biomineralization mechanisms of *Mytilus* and elucidate how it can potentially adapt to predicted global climate changes. Further long-term and possibly multi-generational experimental studies are warranted to quantify the adaptation of *M. coruscus* to predict future climate change.

## Data Availability

The datasets presented in this study can be found in online repositories. The names of the repository/repositories and accession number(s) can be found below: [https://www.ncbi.nlm.nih.gov/bioproject/ and PRJNA918971].

## Supplementary material

SUPPLEMENTARY FIGURE S1 The coverage of reads mapped to *M. coruscus* genome regions. DA, the mussel with drilled shell and raised in acidified sea water (pH 7.4) with exposure time of 48 h; CA, the mussel with complete shell and raised in acidified sea water (pH 7.4) with exposure time of 48 h; DN, the mussel with drilled shell and raised in normal sea water (pH 8.1); CN, the mussel with complete shell and raised in normal sea water (pH 8.1).

SUPPLEMENTARY FIGURE S2 The length distribution of all the assembled transcripts.

SUPPLEMENTARY FIGURE S3 The number of all the assembled transcripts annotated in different databases.

SUPPLEMENTARY FIGURE S4 heatmap of correlation relationship among the 12 mantle samples. DA, the mussel with drilled shell and raised in acidified sea water (pH 7.4) with exposure time of 48 h; CA, the mussel with complete shell and raised in acidified sea water (pH 7.4) with exposure time of 48 h; DN, the mussel with drilled shell and raised in normal sea water (pH 8.1); CN, the mussel with complete shell and raised in normal sea water (pH 8.1).

SUPPLEMENTARY FIGURE S5 Venn map of transcripts from four groups of mantle sample. DA, the mussel with drilled shell and raised in acidified sea water (pH 7.4) with exposure time of 48 h; CA, the mussel with complete shell and raised in acidified sea water (pH 7.4) with exposure time of 48 h; DN, the mussel with drilled shell and raised in normal sea water (pH 8.1); CN, the mussel with complete shell and raised in normal sea water (pH 8.1).

SUPPLEMENTARY FIGURE S6 The volcano maps of DEGs in six pairwise compared samples from mantle. DA, the mussel with drilled shell and raised in acidified sea water (pH 7.4) with exposure time of 48 h; CA, the mussel with complete shell and raised in acidified sea water (pH 7.4) with exposure time of 48 h; DN, the mussel with drilled shell and raised in normal sea water (pH 8.1); CN, the mussel with complete shell and raised in normal sea water (pH 8.1).

SUPPLEMENTARY FIGURE S7 GO annotation of DEGs from six pairwise comparisons of mantle sample. DA, the mussel with drilled shell and raised in acidified sea water (pH 7.4) with exposure time of 48 h; CA, the mussel with complete shell and raised in acidified sea water (pH 7.4) with exposure time of 48 h; DN, the mussel with drilled shell and raised in normal sea water (pH 8.1); CN, the mussel with complete shell and raised in normal sea water (pH 8.1).

SUPPLEMENTARY FIGURE S8 COG annotation of DEGs in six pairwise comparisons of mantle sample. DA, the mussel with drilled shell and raised in acidified sea water (pH 7.4) with exposure time of 48 h; CA, the mussel with complete shell and raised in acidified sea water (pH 7.4) with exposure time of 48 h; DN, the mussel with drilled shell and raised in normal sea water (pH 8.1); CN, the mussel with complete shell and raised in normal sea water (pH 8.1).

SUPPLEMENTARY FIGURE S9 Validation of expression levels of 22 randomly selected transcripts by qPCR comparing with RNA-seq in mantle samples. Correlation analysis was performed using Pearson methods. DA, the mussel with drilled shell and raised in acidified sea water (pH 7.4) with exposure time of 48 h; CA, the mussel with complete shell and raised in acidified sea water (pH 7.4) with exposure time of 48 h; DN, the mussel with drilled shell and raised in normal sea water (pH 8.1); CN, the mussel with complete shell and raised in normal sea water (pH 8.1). The relative expression level was represented by 2^–ΔΔCT^ for qPCR data, and fold change values for RNA-seq data, comparing to the values in CN group.

SUPPLEMENTARY TABLE S1 The primers used for qPCR validation of transcriptome data.

SUPPLEMENTARY TABLE S2 The sequencing statistics of the 12 libraries from mantle samples.

SUPPLEMENTARY TABLE S3 The statistics of clean reads mapped to the *M. coruscus* genome.

SUPPLEMENTARY TABLE S4 The percentage of reads mapped to different genome regions.

SUPPLEMENTARY TABLE S5 Statistics of GO annotated DEGs from six pairwise comparisons of mantle samples. DA, the mussel with drilled shell and raised in acidified sea water (pH 7.4) with exposure time of 48 h; CA, the mussel with complete shell and raised in acidified sea water (pH 7.4) with exposure time of 48 h; DN, the mussel with drilled shell and raised in normal sea water (pH 8.1); CN, the mussel with complete shell and raised in normal sea water (pH 8.1).

SUPPLEMENTARY TABLE S6 TPM values and annotations of biomineralization-related transcripts screened from the transcriptome data. DA, the mussel with drilled shell and raised in acidified sea water (pH 7.4) with exposure time of 48 h; CA, the mussel with complete shell and raised in acidified sea water (pH 7.4) with exposure time of 48 h; DN, the mussel with drilled shell and raised in normal sea water (pH 8.1); CN, the mussel with complete shell and raised in normal sea water (pH 8.1). Only those ranscripts with TPM value >0.01 in each group were listed here.
