# Supplementary material for: Transcriptomic response of Mytilus coruscus mantle to acute sea water acidification and shell damage
Source: Front Physiol. 2023 Oct 26;14:1289655. doi: 10.3389/fphys.2023.1289655 (PMC10639161; doi:10.3389/fphys.2023.1289655)
Supplement: Supplementary file 2 [file Table1.DOCX]

**Supplementary Table 1**

| **Transcript ID** | **Primer-F (5’-3’)** | **Primer-R (5’-3’)** |
| --- | --- | --- |
| M.coruscus_chr5_1516 | AGACGGAATGGAGTAGACCCTCAAG | TTTCGGTGGAACTGGTGGCTTTG |
| M.coruscus_chr14_0948 | CCAGACAATGTTCGCTCGGTGATC | ACAGCCTGGTTGGTTTGTTACGATG |
| M.coruscus_chr11_0352 | GGACCGAAGTGCCCACCATTATG | CTTGGCTGAACTACACCTGACTGAC |
| M.coruscus_chr14_0652 | GCCTACCCTGGAACAACTGAACATC | TCCATTTCAGCTCTGACCAAACCTC |
| M.coruscus_chr12_1649 | AGCATGGAGTCGCAACCAGTTAAG | ACGAATCCACTTGATTGTCCGAGTC |
| M.coruscus_chr3_1738 | GCCAATGAGGATAGCACAGGATGG | GCCCAAGCACTGACTTTCAAATAGC |
| M.coruscus_chr8_1153 | TCGCCTCAGTCAACGGTCTCTAC | ATCATAACCAGTATCGCCAGCCAAG |
| M.coruscus_chr9_2166 | AAAGTGTTCTGTCCCTTCCGTTCG | AGATGACGACCACATTGCGAGAAG |
| M.coruscus_chr13_1761 | CCTAGCACTATGTCGTCTTGTTCCC | TCCTGAAAACCCTCCTACCAGTACC |
| M.coruscus_chr14_1733 | ATCTTCTGGGAGCGGTGGAGTG | CCATTCCGACATCGGCTTCATTCC |
| M.coruscus_chr7_1689 | TCAACAGTCTGTCCCTCCAGTGAG | CTTTGTTCCCCATTCCGACCATACC |
| M.coruscus_chr3_0535 | TTGATTGCTTCCCTGTGTCGTAGC | ACAGCCTTGCCTCTGAAAATAGCC |
| M.coruscus_chr11_1928 | TGGTTCTATGGCAGACGACATTGAC | ATCAGCAGGTTCATTGGCAGATCC |
| M.coruscus_chr1_1710 | AATCCGCACTTACTCTCTGTTTCCC | TGTGGCACATGTAAAGTGGGTGTAG |
| M.coruscus_chr10_0502 | GCAGTTTGACAACAACGGAGAACAC | GAACCTAATATGGCGAGCCCTTCTG |
| M.coruscus_chr1_0493 | GAGGTGATAGGTATGCTGGGAGGAC | TATCTCTTCACTGGCCTGAGTCTGG |
| M.coruscus_chr12_1516 | CGCAGTGGTGGATGTGGAGTTAC | TAGCAACATTGTGACCAGTCCCAAC |
| M.coruscus_chr7_0444 | TGGCAGGATTGAAACCAGCA | TCACTGCTGCTTGTAGATGTTGA |
| M.coruscus_chr8_2680 | AGGCTATGGCAAAAGACAGGAAA | TAGGCCGCATACTGACCTGG |
| M.coruscus_chr3_0057 | CCCACTGAAAGGACGAGGAAACAG | TCATTGGCAGACTTGGCTTTACCC |
| M.coruscus_chr3_1715 | TGGAGCGAGGGGTTGTAAGGAC | CCCGTCGTCTGATAAAGCTGAACAC |
| M.coruscus_chr1_2656 | GTGTCCAGGTCCACAGATTGAAGAG | ACGGGAACACAGGCTACTCCAG |
| EF-1α (internal reference) | CACCACGAGTCTCTCCCTGA | GCTGTCACCACAGACCATTCC |
