# Supplementary material for: Transcriptomic response of Mytilus coruscus mantle to acute sea water acidification and shell damage
Source: Front Physiol. 2023 Oct 26;14:1289655. doi: 10.3389/fphys.2023.1289655 (PMC10639161; doi:10.3389/fphys.2023.1289655)
Supplement: Supplementary file 9 [file Table2.DOCX]

**Supplementary Table 2**

| **Sample** | **Raw reads** | **Raw bases** | **Clean reads** | **Clean bases** | **Error rate (%)** | **Q20(%)** | **Q30(%)** | **GC content (%)** |
| --- | --- | --- | --- | --- | --- | --- | --- | --- |
| CN-1 | 45506070 | 6871416570 | 44138378 | 6554894138 | 0.0266 | 97.35 | 92.79 | 36.26 |
| CN-2 | 46162892 | 6970596692 | 44729258 | 6658904547 | 0.0266 | 97.36 | 92.77 | 35.98 |
| CN-3 | 45817256 | 6918405656 | 44180670 | 6583704399 | 0.0276 | 96.96 | 91.87 | 35.55 |
| CA-1 | 42830432 | 6467395232 | 42094254 | 6229882075 | 0.027 | 97.27 | 92.4 | 35.59 |
| CA-2 | 45200044 | 6825206644 | 44594758 | 6578390018 | 0.0265 | 97.48 | 92.84 | 36.13 |
| CA-3 | 45960876 | 6940092276 | 45285812 | 6704308649 | 0.0267 | 97.37 | 92.6 | 36.93 |
| DN-1 | 45118666 | 6812918566 | 44131928 | 6551999610 | 0.0263 | 97.49 | 92.98 | 36.12 |
| DN-2 | 42185042 | 6369941342 | 41389058 | 6139791377 | 0.0268 | 97.35 | 92.57 | 36.54 |
| DN-3 | 41813314 | 6313810414 | 41149410 | 6074686729 | 0.026 | 97.64 | 93.26 | 36.63 |
| DA-1 | 51558852 | 7785386652 | 50501490 | 7477741327 | 0.0269 | 97.29 | 92.42 | 35.88 |
| DA-2 | 47932098 | 7237746798 | 47245932 | 7003323408 | 0.0264 | 97.5 | 92.96 | 36.24 |
| DA-3 | 42691462 | 6446410762 | 42185898 | 6228655728 | 0.0265 | 97.48 | 92.78 | 36.44 |
