# Supplementary material for: Transcriptomic response of Mytilus coruscus mantle to acute sea water acidification and shell damage
Source: Front Physiol. 2023 Oct 26;14:1289655. doi: 10.3389/fphys.2023.1289655 (PMC10639161; doi:10.3389/fphys.2023.1289655)
Supplement: Supplementary file 10 [file Table5.DOCX]

| **GO ID** | **Description** | **Term Type** | **CA vs. CN percent** | **DA vs. DN percent** | **DA vs. CA percent** | **DN vs. CN percent** | **DA vs. CN percent** | **CA vs. DN percent** |
| --- | --- | --- | --- | --- | --- | --- | --- | --- |
| GO:0032501 | multicellular organismal process | biological_process | 19/3711 | 19/3924 | 1/227 | 6/796 | 11/2744 | 16/3778 |
| GO:0065007 | biological regulation | biological_process | 314/3711 | 343/3924 | 12/227 | 50/796 | 207/2744 | 313/3778 |
| GO:0051704 | multi-organism process | biological_process | 23/3711 | 30/3924 | 4/227 | 6/796 | 17/2744 | 24/3778 |
| GO:0048511 | rhythmic process | biological_process | 2/3711 | 2/3924 | 0 | 1/796 | 1/2744 | 1/3778 |
| GO:0022414 | reproductive process | biological_process | 5/3711 | 9/3924 | 1/227 | 1/796 | 4/2744 | 7/3778 |
| GO:0040011 | locomotion | biological_process | 5/3711 | 3/3924 | 0 | 1/796 | 4/2744 | 2/3778 |
| GO:0098754 | detoxification | biological_process | 1/3711 | 0 | 0 | 0 | 1/2744 | 0 |
| GO:0050896 | response to stimulus | biological_process | 88/3711 | 111/3924 | 5/227 | 21/796 | 67/2744 | 106/3778 |
| GO:0007610 | behavior | biological_process | 1/3711 | 2/3924 | 0 | 0 | 1/2744 | 2/3778 |
| GO:0009987 | cellular process | biological_process | 763/3711 | 825/3924 | 30/227 | 119/796 | 587/2744 | 744/3778 |
| GO:0022610 | biological adhesion | biological_process | 39/3711 | 49/3924 | 8/227 | 13/796 | 29/2744 | 42/3778 |
| GO:0001906 | cell killing | biological_process | 2/3711 | 4/3924 | 0 | 1/796 | 1/2744 | 2/3778 |
| GO:0051179 | localization | biological_process | 126/3711 | 151/3924 | 4/227 | 20/796 | 112/2744 | 135/3778 |
| GO:0032502 | developmental process | biological_process | 35/3711 | 37/3924 | 4/227 | 6/796 | 21/2744 | 31/3778 |
| GO:0002376 | immune system process | biological_process | 41/3711 | 47/3924 | 6/227 | 5/796 | 32/2744 | 41/3778 |
| GO:0071840 | cellular component organization or biogenesis | biological_process | 115/3711 | 108/3924 | 3/227 | 12/796 | 93/2744 | 113/3778 |
| GO:0008152 | metabolic process | biological_process | 541/3711 | 588/3924 | 24/227 | 87/796 | 448/2744 | 546/3778 |
| GO:0023052 | signaling | biological_process | 5/3711 | 3/3924 | 1/227 | 0 | 2/2744 | 3/3778 |
| GO:0040007 | growth | biological_process | 2/3711 | 4/3924 | 2/227 | 0 | 2/2744 | 3/3778 |
| GO:0044425 | membrane part | cellular_component | 1166/3711 | 1307/3924 | 69/227 | 329/796 | 877/2744 | 1231/3778 |
| GO:0044217 | other organism part | cellular_component | 0 | 4/3924 | 0 | 0 | 0 | 2/3778 |
| GO:0044422 | organelle part | cellular_component | 280/3711 | 268/3924 | 7/227 | 33/796 | 214/2744 | 244/3778 |
| GO:0032991 | protein-containing complex | cellular_component | 171/3711 | 179/3924 | 3/227 | 27/796 | 144/2744 | 154/3778 |
| GO:0044464 | cell part | cellular_component | 745/3711 | 797/3924 | 28/227 | 122/796 | 582/2744 | 689/3778 |
| GO:0016020 | membrane | cellular_component | 233/3711 | 279/3924 | 12/227 | 55/796 | 178/2744 | 238/3778 |
| GO:0045202 | synapse | cellular_component | 2/3711 | 3/3924 | 1/227 | 1/796 | 0 | 2/3778 |
| GO:0044456 | synapse part | cellular_component | 11/3711 | 7/3924 | 0 | 5/796 | 5/2744 | 5/3778 |
| GO:0043226 | organelle | cellular_component | 322/3711 | 335/3924 | 7/227 | 49/796 | 245/2744 | 299/3778 |
| GO:0099080 | supramolecular complex | cellular_component | 34/3711 | 18/3924 | 1/227 | 11/796 | 20/2744 | 23/3778 |
| GO:0031974 | membrane-enclosed lumen | cellular_component | 19/3711 | 21/3924 | 0 | 2/796 | 18/2744 | 15/3778 |
| GO:0005576 | extracellular region | cellular_component | 80/3711 | 97/3924 | 11/227 | 45/796 | 62/2744 | 90/3778 |
| GO:0044421 | extracellular region part | cellular_component | 11/3711 | 20/3924 | 0 | 5/796 | 11/2744 | 17/3778 |
| GO:0030054 | cell junction | cellular_component | 3/3711 | 9/3924 | 0 | 2/796 | 3/2744 | 4/3778 |
| GO:0090729 | toxin activity | molecular_function | 4/3711 | 3/3924 | 1/227 | 0 | 5/2744 | 2/3778 |
| GO:0038024 | cargo receptor activity | molecular_function | 14/3711 | 23/3924 | 1/227 | 9/796 | 11/2744 | 18/3778 |
| GO:0005215 | transporter activity | molecular_function | 203/3711 | 205/3924 | 8/227 | 54/796 | 156/2744 | 222/3778 |
| GO:0005488 | binding | molecular_function | 1356/3711 | 1410/3924 | 94/227 | 272/796 | 1022/2744 | 1368/3778 |
| GO:0003824 | catalytic activity | molecular_function | 1148/3711 | 1281/3924 | 68/227 | 201/796 | 883/2744 | 1197/3778 |
| GO:0140104 | molecular carrier activity | molecular_function | 1/3711 | 1/3924 | 0 | 0 | 1/2744 | 1/3778 |
| GO:0005198 | structural molecule activity | molecular_function | 23/3711 | 25/3924 | 2/227 | 3/796 | 17/2744 | 19/3778 |
| GO:0098772 | molecular function regulator | molecular_function | 49/3711 | 77/3924 | 0 | 22/796 | 35/2744 | 61/3778 |
| GO:0045735 | nutrient reservoir activity | molecular_function | 1/3711 | 1/3924 | 0 | 0 | 1/2744 | 1/3778 |
| GO:0016209 | antioxidant activity | molecular_function | 25/3711 | 24/3924 | 3/227 | 7/796 | 17/2744 | 23/3778 |
| GO:0045182 | translation regulator activity | molecular_function | 20/3711 | 17/3924 | 1/227 | 1/796 | 18/2744 | 21/3778 |
| GO:0060089 | molecular transducer activity | molecular_function | 98/3711 | 112/3924 | 10/227 | 22/796 | 69/2744 | 89/3778 |
| GO:0140110 | transcription regulator activity | molecular_function | 72/3711 | 71/3924 | 4/227 | 14/796 | 48/2744 | 68/3778 |
| GO:0044183 | protein folding chaperone | molecular_function | 1/3711 | 1/3924 | 0 | 0 | 1/2744 | 1/3778 |
