# Supplementary material for: Transcriptomic response of Mytilus coruscus mantle to acute sea water acidification and shell damage
Source: Front Physiol. 2023 Oct 26;14:1289655. doi: 10.3389/fphys.2023.1289655 (PMC10639161; doi:10.3389/fphys.2023.1289655)
Supplement: Supplementary file 11 [file Table3.DOCX]

**Supplementary Table 3**

| **Sample** | **Total reads** | **Total mapped** | **Ratio** | **Multiple mapped** | **Ratio** | **Uniquely mapped** | **Ratio** |
| --- | --- | --- | --- | --- | --- | --- | --- |
| CN-1 | 44138378 | 27858456 | 63.12% | 1914027 | 2.07% | 26944429 | 61.05% |
| CN-2 | 44729258 | 29320896 | 65.55% | 1919970 | 4.29% | 28400926 | 63.50% |
| CN-3 | 44180670 | 29417865 | 66.59% | 1455056 | 3.29% | 22962809 | 51.97% |
| CA-1 | 42094254 | 29720261 | 70.60% | 1953555 | 4.64% | 28766706 | 68.34% |
| CA-2 | 44594758 | 28187988 | 63.21% | 2395249 | 5.37% | 26792739 | 60.08% |
| CA-3 | 45285812 | 25212769 | 55.67% | 2329974 | 5.15% | 23882795 | 52.74% |
| DN-1 | 44131928 | 24639889 | 55.83% | 2282931 | 5.17% | 23356958 | 52.93% |
| DN-2 | 41389058 | 25214888 | 60.92% | 2283084 | 5.52% | 23931804 | 57.82% |
| DN-3 | 41149410 | 24570140 | 59.71% | 2227892 | 5.41% | 23342248 | 56.73% |
| DA-1 | 50501490 | 26726350 | 52.92% | 1768439 | 3.50% | 25957911 | 51.40% |
| DA-2 | 47245932 | 24766483 | 52.42% | 1726030 | 3.65% | 24040453 | 50.88% |
| DA-3 | 42185898 | 26359304 | 62.48% | 2348954 | 5.57% | 25010350 | 59.29% |
