# Supplementary material for: Transcriptomic response of Mytilus coruscus mantle to acute sea water acidification and shell damage
Source: Front Physiol. 2023 Oct 26;14:1289655. doi: 10.3389/fphys.2023.1289655 (PMC10639161; doi:10.3389/fphys.2023.1289655)
Supplement: Supplementary file 12 [file Table4.DOCX]

**Supplementary Table 4**

| **Sample** | **CDS (percentage)** | **Intergenic (percentage)** | **Introns (percentage)** |
| --- | --- | --- | --- |
| CN-1 | 19005647.0(70.78%) | 5138049.0(19.14%) | 2707653.0(10.08%) |
| CN-2 | 19759271.0(68.56%) | 5929531.0(20.57%) | 3132337.0(10.87%) |
| CN-3 | 10125274.0(70.8%) | 2740766.0(19.17%) | 1434649.0(10.03%) |
| CA-1 | 16816562.0(60.95%) | 7195039.0(26.08%) | 3580828.0(12.98%) |
| CA-2 | 25124037.0(63.17%) | 9355639.0(23.52%) | 5291754.0(13.31%) |
| CA-3 | 24799950.0(66.4%) | 7744998.0(20.74%) | 4806362.0(12.87%) |
| DN-1 | 24275039.0(67.26%) | 7723231.0(21.4%) | 4091767.0(11.34%) |
| DN-2 | 24944580.0(67.84%) | 7649032.0(20.8%) | 4176202.0(11.36%) |
| DN-3 | 24910316.0(68.9%) | 7309240.0(20.22%) | 3934509.0(10.88%) |
| DA-1 | 15873942.0(64.32%) | 5031970.0(20.39%) | 3772079.0(15.29%) |
| DA-2 | 15435714.0(69.4%) | 4222085.0(18.98%) | 2583339.0(11.62%) |
| DA-3 | 23954312.0(64.43%) | 8256818.0(22.21%) | 4964825.0(13.35%) |
