# Supplementary material for: Transcriptomic response of Mytilus coruscus mantle to acute sea water acidification and shell damage
Source: Front Physiol. 2023 Oct 26;14:1289655. doi: 10.3389/fphys.2023.1289655 (PMC10639161; doi:10.3389/fphys.2023.1289655)
Supplement: Supplementary file 14 [file Table6.DOCX]

| **Gene ID** | **CN (TPM)** | **DN (TPM)** | **CA (TPM)** | **DA (TPM)** | **NR ID** | **NR description [Species]** | **Swiss-Prot hit ID** | **Swiss-Prot description** |
| --- | --- | --- | --- | --- | --- | --- | --- | --- |
| **Shell proteins** |  |  |  |  |  |  |  |  |
| M.coruscus_chr11_2675 | 1.06 | 3.47 | 11.98 | 14.52 | AKI87975.1 | shell protein-4 [Mytilus coruscus] | sp\|Q17043\|APLY_APLKU; | Aplysianin-A OS=Aplysia kurodai OX=6501 PE=1 SV=1; |
| M.coruscus_chr14_1543 | 2.18 | 4.82 | 9.03 | 9.38 | AKI87977.1 | shell protein-6 [Mytilus coruscus] | sp\|H2A0L8\|FND2_PINMG; | Fibronectin type III domain-containing protein 2 OS=Margaritifera margaritifera OX=102329 PE=1 SV=1; |
| M.coruscus_chr2_2414 | 7.70 | 5.57 | 51.03 | 33.56 | AKI87979.1 | shell mytilin-2 [Mytilus coruscus] | sp\|P86858\|MYT2_MYTCA; | Mytilin-2 OS=Mytilus californianus OX=6549 PE=1 SV=1; |
| M.coruscus_chr4_0657 | 0.24 | 0.26 | 4.36 | 2.55 | CAC5380900.1 | Shell matrix protein [Mytilus coruscus] | sp\|P86860\|PSM_MYTCA; | Shell matrix protein; |
| M.coruscus_chr7_1920 | 3.83 | 22.22 | 13.05 | 12.90 | CAC5419280.1 | Shell matrix protein | sp\|P86860\|PSM_MYTCA; | Shell matrix protein; |
| M.coruscus_chr8_2420 | 7.62 | 16.40 | 49.84 | 34.55 | AKI87980.1 | shell mytilin-3 [Mytilus coruscus] | sp\|P86859\|MYT3_MYTCA; | Mytilin-3 OS=Mytilus californianus OX=6549 PE=1 SV=1; |
| M.coruscus_chr8_2440 | 61.31 | 706.17 | 2260.33 | 844.36 | AKI87972.1 | shell protein-1 [Mytilus coruscus] | ------ | ------ |
| M.coruscus_chr9_1432 | 4.57 | 19.27 | 33.10 | 37.70 | AKI87973.1 | shell protein-2 [Mytilus coruscus] | ------ | ------ |
| **Nacrein** |  |  |  |  |  |  |  |  |
| M.coruscus_chr7_1681 | 7.19 | 13.85 | 88.08 | 83.73 | AKS48162.1 | Nacrein-like protein-2 [Mytilus coruscus] | sp\|P86856\|MANL_MYTCA; | Nacrein-like protein; |
| **EP-protein** |  |  |  |  |  |  |  |  |
| M.coruscus_chr8_2671 | 129.65 | 49.26 | 129.11 | 111.53 | AKS48159.1 | EP-protein-1 [Mytilus coruscus] | sp\|P83425\|HIP_MYTED; | Heavy metal-binding protein HIP OS=Mytilus edulis OX=6550 PE=1 SV=1; |
| **Sushi-like protein** |  |  |  |  |  |  |  |  |
| M.coruscus_chr7_1956 | 2.84 | 7.00 | 12.28 | 13.50 | AKS48157.1 | Sushi-like protein [Mytilus coruscus] | sp\|H2A0N4\|PIF_PINMG; | Protein PIF OS=Margaritifera margaritifera OX=102329 PE=1 SV=1; |
| **Lustrin** |  |  |  |  |  |  |  |  |
| M.coruscus_chr11_0349 | 14.90 | 240.54 | 26.56 | 61.00 | CAC5397517.1 | Lustrin like | sp\|P09412\|WAP_RABIT; | Whey acidic protein OS=Oryctolagus cuniculus OX=9986 GN=WAP PE=2 SV=1; |
| M.coruscus_chr11_0352 | 4.49 | 1940.40 | 56.60 | 113.15 | CAC5397525.1 | Lustrin like | sp\|P00993\|IBP_CARCR; | Chelonianin OS=Caretta caretta OX=8467 PE=1 SV=1; |
| **Perlwapin** |  |  |  |  |  |  |  |  |
| M.coruscus_chr11_1797 | 8.63 | 81.81 | 11.39 | 1.75 | CAC5399421.1 | Perlwapin-like protein | sp\|P86855\|PWAPL_MYTGA; | Perlwapin-like protein OS=Mytilus galloprovincialis OX=29158 PE=1 SV=1; |
| **Regucalcin** |  |  |  |  |  |  |  |  |
| M.coruscus_chr13_0626 | 3.32 | 3.39 | 7.90 | 8.82 | CAC5376883.1 | Regucalcin | sp\|Q6DF62\|RGN_XENTR; | Regucalcin OS=Xenopus tropicalis OX=8364 GN=rgn PE=2 SV=1; |
| **Carbonic anhydrase** |  |  |  |  |  |  |  |  |
| M.coruscus_chr13_1329 | 11.70 | 5.70 | 9.48 | 5.54 | CAC5401222.1 | CA [Mytilus coruscus] | sp\|P00918\|CAH2_HUMAN; | Carbonic anhydrase 2 OS=Homo sapiens OX=9606 GN=CA2 PE=1 SV=2; |
| M.coruscus_chr1_2987 | 33.99 | 79.91 | 44.91 | 20.26 | CAC5399079.1 | TPMT [Mytilus coruscus] | sp\|Q504A5\|TPMT_DANRE; | Probable thiopurine S-methyltransferase OS=Danio rerio OX=7955 GN=tpmt PE=2 SV=1; |
| M.coruscus_chr1_2989 | 0.22 | 0.72 | 2.36 | 0.77 | CAC5399080.1 | CA [Mytilus coruscus] | sp\|P48283\|CAH4_RABIT; | Carbonic anhydrase 4 OS=Oryctolagus cuniculus OX=9986 GN=CA4 PE=1 SV=1; |
| M.coruscus_chr2_2533 | 7.86 | 5.92 | 8.14 | 9.02 | CAC5378916.1 | CA [Mytilus coruscus] | sp\|Q6P0I8\|EXOS6_DANRE; | Exosome complex component MTR3 OS=Danio rerio OX=7955 GN=exosc6 PE=2 SV=2; |
| M.coruscus_chr2_3250 | 0.52 | 2.83 | 4.36 | 2.83 | CAC5401931.1 | SLC28A [Mytilus coruscus] | sp\|Q9HAS3\|S28A3_HUMAN; | Solute carrier family 28 member 3 OS=Homo sapiens OX=9606 GN=SLC28A3 PE=1 SV=1; |
| M.coruscus_chr4_0064 | 0.44 | 0.60 | 0.86 | 0.76 | CAG2190362.1 | unnamed protein product [Mytilus edulis] | sp\|Q18932\|CAH2_CAEEL; | Putative carbonic anhydrase-like protein 2 OS=Caenorhabditis elegans OX=6239 GN=cah-2 PE=1 SV=3; |
| M.coruscus_chr4_0773 | 1.04 | 3.21 | 2.03 | 1.72 | CAC5374312.1 | unnamed protein product [Mytilus coruscus] | sp\|A0JN41\|CAH10_BOVIN; | Carbonic anhydrase-related protein 10 OS=Bos taurus OX=9913 GN=CA10 PE=2 SV=1; |
| M.coruscus_chr4_2425 | 2.01 | 2.10 | 3.05 | 1.78 | CAC5396389.1 | unnamed protein product [Mytilus coruscus] | sp\|A0JN41\|CAH10_BOVIN; | Carbonic anhydrase-related protein 10 OS=Bos taurus OX=9913 GN=CA10 PE=2 SV=1; |
| M.coruscus_chr5_0800 | 14.41 | 6.50 | 3.71 | 9.71 | CAC5392222.1 | CA2 [Mytilus coruscus] | sp\|Q8UWA5\|CAH2_TRIHK; | Carbonic anhydrase 2 OS=Tribolodon hakonensis OX=151740 GN=ca2 PE=2 SV=3; |
| M.coruscus_chr5_0801 | 6.26 | 3.25 | 2.46 | 11.95 | CAG2245110.1 | CA2 [Mytilus edulis] | sp\|Q92051\|CAHZ_DANRE; | Carbonic anhydrase OS=Danio rerio OX=7955 GN=cahz PE=1 SV=2; |
| M.coruscus_chr5_2670 | 0.21 | 0.75 | 1.09 | 3.25 | VDI28625.1 | carbonic anhydrase [Mytilus galloprovincialis] | sp\|P28651\|CAH8_MOUSE; | Carbonic anhydrase-related protein OS=Mus musculus OX=10090 GN=Ca8 PE=1 SV=5; |
| M.coruscus_chr7_0094 | 2.15 | 2.52 | 3.35 | 2.69 | CAG2187981.1 | unnamed protein product [Mytilus edulis] | sp\|P00917\|CAH1_HORSE; | Carbonic anhydrase 1 OS=Equus caballus OX=9796 GN=CA1 PE=1 SV=3; |
| M.coruscus_chr7_2254 | 0.17 | 0.93 | 1.53 | 1.81 | CAC5379890.1 | CA [Mytilus coruscus] | sp\|Q9MZ30\|CAH12_RABIT; | Carbonic anhydrase 12 OS=Oryctolagus cuniculus OX=9986 GN=CA12 PE=2 SV=2; |
| M.coruscus_chr7_2434 | 6.36 | 22.33 | 25.98 | 40.00 | CAC5361125.1 | CA [Mytilus coruscus] | sp\|Q8CI85\|CAH12_MOUSE; | Carbonic anhydrase 12 OS=Mus musculus OX=10090 GN=Ca12 PE=1 SV=1; |
| M.coruscus_chr7_2446 | 15.67 | 22.34 | 85.90 | 63.98 | CAC5361125.1 | CA [Mytilus coruscus] | sp\|Q8CI85\|CAH12_MOUSE; | Carbonic anhydrase 12 OS=Mus musculus OX=10090 GN=Ca12 PE=1 SV=1; |
| M.coruscus_chr7_2690 | 2.44 | 5.39 | 18.45 | 23.71 | VDI18017.1 | carbonic anhydrase [Mytilus galloprovincialis] | sp\|Q9WVT6\|CAH14_MOUSE; | Carbonic anhydrase 14 OS=Mus musculus OX=10090 GN=Ca14 PE=1 SV=1; |
| M.coruscus_chr7_2691 | 0.91 | 1.30 | 7.81 | 13.08 | CAC5377554.1 | CA [Mytilus coruscus] | sp\|Q9WVT6\|CAH14_MOUSE; | Carbonic anhydrase 14 OS=Mus musculus OX=10090 GN=Ca14 PE=1 SV=1; |
| M.coruscus_chr8_1513 | 2.00 | 10.07 | 3.58 | 3.41 | CAG2243449.1 | CA [Mytilus edulis] | sp\|Q9ULX7\|CAH14_HUMAN; | Carbonic anhydrase 14 OS=Homo sapiens OX=9606 GN=CA14 PE=1 SV=1; |
| **Perlucin** |  |  |  |  |  |  |  |  |
| M.coruscus_chr10_1227 | 225.53 | 198.26 | 69.08 | 75.88 | VDI35066.1 | Hypothetical predicted protein [Mytilus galloprovincialis] | sp\|P86854\|PLCL_MYTGA; | Perlucin-like protein OS=Mytilus galloprovincialis OX=29158 PE=1 SV=1; |
| M.coruscus_chr11_0626 | 0.37 | 0.32 | 1.10 | 2.16 | CAC5386980.1 | COLEC12 [Mytilus coruscus] | sp\|P86854\|PLCL_MYTGA; | Perlucin-like protein OS=Mytilus galloprovincialis OX=29158 PE=1 SV=1; |
| M.coruscus_chr12_1569 | 95.91 | 319.68 | 44.33 | 208.38 | CAC5414330.1 | unnamed protein product [Mytilus coruscus] | sp\|P82596\|PLC_HALLA; | Perlucin OS=Haliotis laevigata OX=36097 PE=1 SV=3; |
| M.coruscus_chr12_1857 | 7.60 | 5.13 | 7.14 | 2.48 | CAC5406223.1 | unnamed protein product [Mytilus coruscus] | sp\|P86854\|PLCL_MYTGA; | Perlucin-like protein OS=Mytilus galloprovincialis OX=29158 PE=1 SV=1; |
| M.coruscus_chr13_1401 | 1.53 | 1.39 | 3.17 | 4.11 | CAC5371921.1 | unnamed protein product [Mytilus coruscus] | sp\|P82596\|PLC_HALLA; | Perlucin OS=Haliotis laevigata OX=36097 PE=1 SV=3; |
| M.coruscus_chr1_0490 | 1.42 | 0.22 | 0.96 | 0.80 | CAC5361411.1 | MRC [Mytilus coruscus] | sp\|P86854\|PLCL_MYTGA; | Perlucin-like protein OS=Mytilus galloprovincialis OX=29158 PE=1 SV=1; |
| M.coruscus_chr2_1415 | 30.84 | 37.74 | 15.23 | 6.47 | CAC5388321.1 | MRC [Mytilus coruscus] | sp\|P86854\|PLCL_MYTGA; | Perlucin-like protein OS=Mytilus galloprovincialis OX=29158 PE=1 SV=1; |
| M.coruscus_chr2_3351 | 9.99 | 98.32 | 2.77 | 1.92 | CAC5382595.1 | unnamed protein product [Mytilus coruscus] | sp\|P86854\|PLCL_MYTGA; | Perlucin-like protein OS=Mytilus galloprovincialis OX=29158 PE=1 SV=1; |
| M.coruscus_chr2_3357 | 24.15 | 355.91 | 3.85 | 6.35 | CAC5382595.1 | unnamed protein product [Mytilus coruscus] | sp\|P86854\|PLCL_MYTGA; | Perlucin-like protein OS=Mytilus galloprovincialis OX=29158 PE=1 SV=1; |
| M.coruscus_chr7_0322 | 1.70 | 0.86 | 12.70 | 27.15 | CAC5362301.1 | unnamed protein product [Mytilus coruscus] | sp\|P86854\|PLCL_MYTGA; | Perlucin-like protein OS=Mytilus galloprovincialis OX=29158 PE=1 SV=1; |
| M.coruscus_chr7_0736 | 0.43 | 2.23 | 14.31 | 76.55 | CAC5415889.1 | unnamed protein product [Mytilus coruscus] | sp\|P86854\|PLCL_MYTGA; | Perlucin-like protein OS=Mytilus galloprovincialis OX=29158 PE=1 SV=1; |
| M.coruscus_chr8_2457 | 2.20 | 1.02 | 0.74 | 3.27 | VDI13019.1 | Hypothetical predicted protein [Mytilus galloprovincialis] | sp\|P82596\|PLC_HALLA; | Perlucin OS=Haliotis laevigata OX=36097 PE=1 SV=3; |
| M.coruscus_chr9_0050 | 7.95 | 8.93 | 11.77 | 6.88 | CAC5383112.1 | unnamed protein product [Mytilus coruscus] | sp\|P86854\|PLCL_MYTGA; | Perlucin-like protein OS=Mytilus galloprovincialis OX=29158 PE=1 SV=1; |
| M.coruscus_chr9_0232 | 3.99 | 4.12 | 2.84 | 2.58 | CAC5418060.1 | unnamed protein product [Mytilus coruscus] | sp\|P86854\|PLCL_MYTGA; | Perlucin-like protein OS=Mytilus galloprovincialis OX=29158 PE=1 SV=1; |
| **Tyrosinase** |  |  |  |  |  |  |  |  |
| M.coruscus_chr10_1292 | 3.80 | 21.74 | 1.73 | 12.33 | CAC5385294.1 | TYR [Mytilus coruscus] | sp\|P86952\|TYRO_PINMA; | Tyrosinase-like protein OS=Pinctada maxima OX=104660 PE=1 SV=1; |
| M.coruscus_chr13_0036 | 0.20 | 6.32 | 2.57 | 1.80 | CAC5404578.1 | unnamed protein product [Mytilus coruscus] | sp\|H2A0L1\|TYRO2_PINMG; | Tyrosinase-like protein 2 OS=Margaritifera margaritifera OX=102329 PE=1 SV=1; |
| M.coruscus_chr13_0628 | 0.50 | 3.54 | 5.66 | 6.37 | CAC5376890.1 | unnamed protein product [Mytilus coruscus] | sp\|H2A0L1\|TYRO2_PINMG; | Tyrosinase-like protein 2 OS=Margaritifera margaritifera OX=102329 PE=1 SV=1; |
| M.coruscus_chr2_0118 | 3.95 | 66.26 | 119.31 | 73.14 | CAC5424132.1 | unnamed protein product [Mytilus coruscus] | sp\|H2A0L0\|TYRO1_PINMG; | Tyrosinase-like protein 1 OS=Margaritifera margaritifera OX=102329 PE=1 SV=1; |
| M.coruscus_chr5_0837 | 0.45 | 2.08 | 5.57 | 6.56 | CAC5409139.1 | unnamed protein product [Mytilus coruscus] | sp\|H2A0L0\|TYRO1_PINMG; | Tyrosinase-like protein 1 OS=Margaritifera margaritifera OX=102329 PE=1 SV=1; |
| M.coruscus_chr5_0839 | 0.24 | 0.74 | 5.05 | 2.35 | CAC5409142.1 | TYR [Mytilus coruscus] | sp\|H2A0L0\|TYRO1_PINMG; | Tyrosinase-like protein 1 OS=Margaritifera margaritifera OX=102329 PE=1 SV=1; |
| M.coruscus_chr5_1855 | 13.66 | 9.12 | 11.78 | 11.57 | VDI29348.1 | WD repeat-containing protein 59 [Mytilus galloprovincialis] | sp\|Q6PJI9\|WDR59_HUMAN; | GATOR complex protein WDR59 OS=Homo sapiens OX=9606 GN=WDR59 PE=1 SV=2; |
| M.coruscus_chr5_1878 | 3.51 | 17.80 | 47.58 | 30.64 | CAC5423994.1 | unnamed protein product [Mytilus coruscus] | sp\|Q19673\|TYR3_CAEEL; | Putative tyrosinase-like protein tyr-3 OS=Caenorhabditis elegans OX=6239 GN=tyr-3 PE=3 SV=5; |
| M.coruscus_chr5_2258 | 3.68 | 9.54 | 18.32 | 14.53 | CAG2214400.1 | unnamed protein product [Mytilus edulis] | sp\|P34269\|TYR1_CAEEL; | Putative tyrosinase-like protein tyr-1 OS=Caenorhabditis elegans OX=6239 GN=tyr-1 PE=1 SV=2; |
| **Chitinase** |  |  |  |  |  |  |  |  |
| M.coruscus_chr10_0199 | 0.35 | 0.33 | 0.47 | 0.61 | CAC5409184.1 | E3.2.1.14 [Mytilus coruscus] | sp\|Q9W5U2\|CHI10_DROME; | Probable chitinase 10 OS=Drosophila melanogaster OX=7227 GN=Cht10 PE=2 SV=2; |
| M.coruscus_chr10_0206 | 2.55 | 6.85 | 7.82 | 6.86 | CAC5409184.1 | E3.2.1.14 [Mytilus coruscus] | sp\|Q9W5U2\|CHI10_DROME; | Probable chitinase 10 OS=Drosophila melanogaster OX=7227 GN=Cht10 PE=2 SV=2; |
| M.coruscus_chr10_0416 | 32.05 | 48.91 | 18.74 | 27.84 | VDI65885.1 | chitinase, partial [Mytilus galloprovincialis] | sp\|Q9W5U2\|CHI10_DROME; | Probable chitinase 10 OS=Drosophila melanogaster OX=7227 GN=Cht10 PE=2 SV=2; |
| M.coruscus_chr10_0429 | 3.24 | 5.25 | 5.20 | 3.06 | CAC5392475.1 | E3.2.1.14 [Mytilus coruscus] | sp\|Q6RY07\|CHIA_RAT; | Acidic mammalian chitinase OS=Rattus norvegicus OX=10116 GN=Chia PE=2 SV=1; |
| M.coruscus_chr10_0533 | 112.65 | 168.47 | 106.20 | 109.02 | CAC5402166.1 | PCDH15 [Mytilus coruscus] | sp\|H2A0L4\|CHI1_PINMG; | Putative chitinase 1 OS=Margaritifera margaritifera OX=102329 PE=1 SV=1; |
| M.coruscus_chr10_0883 | 4.31 | 5.90 | 4.30 | 2.79 | CAC5375846.1 | E3.2.1.14 [Mytilus coruscus] | sp\|Q9W5U2\|CHI10_DROME; | Probable chitinase 10 OS=Drosophila melanogaster OX=7227 GN=Cht10 PE=2 SV=2; |
| M.coruscus_chr10_0885 | 2.02 | 4.29 | 0.02 | 1.46 | CAC5375846.1 | E3.2.1.14 [Mytilus coruscus] | sp\|Q6RY07\|CHIA_RAT; | Acidic mammalian chitinase OS=Rattus norvegicus OX=10116 GN=Chia PE=2 SV=1; |
| M.coruscus_chr10_1198 | 3.09 | 13.71 | 10.23 | 8.58 | CAC5377702.1 | E3.2.1.14 [Mytilus coruscus] | sp\|Q9W5U2\|CHI10_DROME; | Probable chitinase 10 OS=Drosophila melanogaster OX=7227 GN=Cht10 PE=2 SV=2; |
| M.coruscus_chr11_0630 | 20.60 | 3.99 | 30.08 | 5.03 | CAG2229341.1 | unnamed protein product [Mytilus edulis] | sp\|P27115\|MGAT1_RABIT; | Alpha-1,3-mannosyl-glycoprotein 2-beta-N-acetylglucosaminyltransferase OS=Oryctolagus cuniculus OX=9986 GN=MGAT1 PE=1 SV=1; |
| M.coruscus_chr2_3224 | 41.23 | 50.10 | 23.35 | 26.30 | CAC5401905.1 | E3.2.1.14 [Mytilus coruscus] | sp\|Q15782\|CH3L2_HUMAN; | Chitinase-3-like protein 2 OS=Homo sapiens OX=9606 GN=CHI3L2 PE=1 SV=1; |
| M.coruscus_chr8_2573 | 2.50 | 4.27 | 7.62 | 6.72 | CAC5418621.1 | CHID1 [Mytilus coruscus] | sp\|Q66IL0\|CHID1_XENTR; | Chitinase domain-containing protein 1 OS=Xenopus tropicalis OX=8364 GN=chid1 PE=2 SV=1; |
| **Chitin synthase** |  |  |  |  |  |  |  |  |
| M.coruscus_chr11_0844 | 1.67 | 4.30 | 5.80 | 3.57 | CAC5426140.1 | CHS1 [Mytilus coruscus] | sp\|G5EBQ8\|CHS2_CAEEL; | Chitin synthase chs-2 OS=Caenorhabditis elegans OX=6239 GN=chs-2 PE=1 SV=1; |
| M.coruscus_chr11_0845 | 7.78 | 3.90 | 9.18 | 5.13 | CAC5426141.1 | CHS1 [Mytilus coruscus] | sp\|G5EBQ8\|CHS2_CAEEL; | Chitin synthase chs-2 OS=Caenorhabditis elegans OX=6239 GN=chs-2 PE=1 SV=1; |
| M.coruscus_chr14_0895 | 1.69 | 0.93 | 2.09 | 4.31 | VDI58933.1 | chitin synthase [Mytilus galloprovincialis] | sp\|G5EBQ8\|CHS2_CAEEL; | Chitin synthase chs-2 OS=Caenorhabditis elegans OX=6239 GN=chs-2 PE=1 SV=1; |
| M.coruscus_chr14_1365 | 1.62 | 6.38 | 12.35 | 13.14 | CAC5377820.1 | CHS1 [Mytilus coruscus] | sp\|G5EBQ8\|CHS2_CAEEL; | Chitin synthase chs-2 OS=Caenorhabditis elegans OX=6239 GN=chs-2 PE=1 SV=1; |
| M.coruscus_chr2_2136 | 5.41 | 9.65 | 20.09 | 14.39 | CAC5419944.1 | CHS1 [Mytilus coruscus] | sp\|G5EBQ8\|CHS2_CAEEL; | Chitin synthase chs-2 OS=Caenorhabditis elegans OX=6239 GN=chs-2 PE=1 SV=1; |
| M.coruscus_chr2_3088 | 0.27 | 0.73 | 10.23 | 5.50 | CAC5405890.1 | CHS1 [Mytilus coruscus] | sp\|G5EBQ8\|CHS2_CAEEL; | Chitin synthase chs-2 OS=Caenorhabditis elegans OX=6239 GN=chs-2 PE=1 SV=1; |
| M.coruscus_chr4_0657 | 0.24 | 0.26 | 4.36 | 2.55 | CAC5380900.1 | Shell matrix protein [Mytilus coruscus] | sp\|P86860\|PSM_MYTCA; | Shell matrix protein; |
| M.coruscus_chr6_2294 | 3.21 | 1.79 | 2.63 | 3.93 | VDI02444.1 | chitin synthase [Mytilus galloprovincialis] | sp\|G5EBQ8\|CHS2_CAEEL; | Chitin synthase chs-2 OS=Caenorhabditis elegans OX=6239 GN=chs-2 PE=1 SV=1; |
| M.coruscus_chr6_2554 | 0.80 | 2.29 | 1.08 | 0.79 | CAC5424960.1 | unnamed protein product [Mytilus coruscus] | sp\|H9J9M0\|CDA1_BOMMO; | Chitin deacetylase 1 OS=Bombyx mori OX=7091 GN=CDA1 PE=1 SV=1; |
| M.coruscus_chr8_0295 | 0.12 | 0.35 | 0.64 | 0.47 | CAC5403722.1 | unnamed protein product [Mytilus coruscus] | sp\|H9J9M0\|CDA1_BOMMO; | Chitin deacetylase 1 OS=Bombyx mori OX=7091 GN=CDA1 PE=1 SV=1; |
| M.coruscus_chr8_0394 | 0.13 | 0.90 | 3.19 | 2.04 | CAC5380821.1 | unnamed protein product [Mytilus coruscus] | sp\|H9JW44\|CDA7_BOMMO; | Chitin deacetylase 7 OS=Bombyx mori OX=7091 GN=CDA7 PE=1 SV=1; |
| M.coruscus_chr8_1287 | 0.64 | 1.12 | 5.09 | 3.47 | CAC5394645.1 | CHS1 [Mytilus coruscus] | sp\|G5EBQ8\|CHS2_CAEEL; | Chitin synthase chs-2 OS=Caenorhabditis elegans OX=6239 GN=chs-2 PE=1 SV=1; |
| M.coruscus_chr8_1290 | 0.56 | 2.14 | 13.95 | 10.63 | ABQ08059.1 | chitin synthase [Mytilus galloprovincialis] | sp\|G5EBQ8\|CHS2_CAEEL; | Chitin synthase chs-2 OS=Caenorhabditis elegans OX=6239 GN=chs-2 PE=1 SV=1; |
| M.coruscus_chr8_1520 | 2.15 | 5.48 | 11.46 | 8.45 | CAC5411437.1 | CHS1 [Mytilus coruscus] | sp\|G5EBQ8\|CHS2_CAEEL; | Chitin synthase chs-2 OS=Caenorhabditis elegans OX=6239 GN=chs-2 PE=1 SV=1; |
